# AI-Enhanced Directional Pedestrian Sensing Using a Single MEMS Accelerometer

**DOI:** 10.3390/s26185736

**Published:** 2026-09-09

**Authors:** Enric Casademont, Narcís Planellas, Carles Pous, Llorenç Burgas, Joaquim Massana, Pere Marti-Puig

**Affiliations:** 1 Department of Electrical, Electronic and Automatic Control Engineering, University of Girona, 17003 Girona, Spain; narcis.planellas@udg.edu (N.P.); carles.pous@udg.edu (C.P.); llorenc.burgas@udg.edu (L.B.); joaquim.massana@udg.edu (J.M.); 2 Data and Signal Processing Group, University of Vic—Central University of Catalonia, 08500 Vic, Spain

**Keywords:** MEMS accelerometer, directional sensing, pedestrian flow monitoring, vibration-based sensing, single-sensor system, industrial sensors, machine learning

## Abstract

Artificial intelligence can extend the functional capabilities of embedded sensors by extracting application-level information from physical measurements. This study investigates whether footstep-induced floor vibrations acquired with a single triaxial MEMS accelerometer contain sufficient information to characterize pedestrian path orientation and travel sense. A custom sensing platform based on an ADXL355 accelerometer and an ESP32 microcontroller was developed to acquire the structural vibration response at 4 kSPS. Lightweight temporal features were processed using a Random Forest classifier. The primary assessment used leakage-aware event-level cross-validation, with complete footsteps as the data-partitioning units. Under this more conservative protocol, discrimination of the complete A–K movement-label set was poor, whereas a compact 12-dimensional descriptor representation achieved 73.18% accuracy, 71.52% balanced accuracy, and 70.98% macro-F1 for X/Y path-orientation classification. Reliable positive/negative travel-sense discrimination could not be demonstrated from isolated footsteps. For historical comparison, the original sample-level procedure yielded 97.09% accuracy, but this value is retained only as a within-sequence reference because densely sampled observations contain strongly overlapping information. The findings provide proof-of-concept evidence that a single floor-mounted MEMS accelerometer can capture coarse pedestrian path-orientation information without cameras or spatially distributed vibration-sensor networks. Feature extraction and classifier inference were performed offline; broader validation across participants, sessions, floor structures, and realistic disturbances is required before deployment as an embedded edge AI sensing node.

## 1. Introduction

The increasing integration of artificial intelligence (AI), embedded electronics, and Industrial Internet of Things (IIoT) technologies is transforming conventional measurement devices into smart sensing systems capable of extracting application-level information from physical signals. This evolution is consistent with the Industry 5.0 paradigm, which places human well-being, safety, and human-centric technology at the center of industrial development [1,2].

In human-centric industrial environments, reliable knowledge of pedestrian presence, movement direction, and flow can support safety supervision, access management, workspace utilization, logistics, emergency evacuation, and the adaptive operation of lighting, ventilation, and other building services. Similar information is relevant in smart buildings, where occupancy-aware monitoring and control can contribute to reducing energy consumption while maintaining user comfort [3,4,5,6].

Pedestrian-monitoring systems intended for industrial and public environments must satisfy several requirements simultaneously. They should provide timely and sufficiently detailed information, preserve user privacy, remain affordable, and require minimal modifications to the existing infrastructure. Conventional sensing technologies only partially meet these requirements. Passive infrared (PIR) sensors are inexpensive and widely deployed but generally provide only motion or presence information and cannot reliably determine pedestrian direction or flow. Carbon dioxide sensors provide an indirect and comparatively slow estimate of occupancy. Camera-based systems can offer accurate counting and tracking, but their deployment may be constrained by privacy concerns, lighting conditions, occlusion, installation complexity, data-storage requirements, and computational cost. Pressure mats provide accurate local passage detection, but their coverage is limited and their integration into the floor can be intrusive [3,6,7].

Table 1 summarizes the main functional capabilities and deployment constraints of representative pedestrian-monitoring technologies. Rather than assigning general qualitative ratings such as high or low accuracy, the comparison focuses on the information that each sensing principle can provide and the infrastructure required for its deployment.

Vibration-based sensing provides an alternative way to detect human activity without directly observing or identifying individuals. Human footsteps generate impulsive forces that excite structural vibrations in floors. These vibrations propagate through the structure and can be measured using accelerometers, geophones, or piezoelectric transducers [7,8,9,10,11,12,13,14]. Since the sensing principle relies on the mechanical response of the floor rather than on images, wearable devices, or actively transmitted signals, it is device-free and generally perceived as privacy-preserving. Furthermore, a floor-mounted vibration sensor can monitor passage events over an area larger than the immediate sensing surface of a pressure mat or other localized detector.

Footstep-induced structural vibrations contain information at different temporal and spectral scales. Their characteristics depend on the force applied during foot–floor contact and on factors such as walking speed, footwear, body characteristics, sensor distance, floor material, and structural layout [15,16,17,18]. The structure therefore acts both as a propagation medium and as part of the measurement system. Recent datasets covering different floor types, sensor distances, and walking conditions further illustrate the strong dependence of structural-vibration signatures on both the pedestrian and the measurement environment [13]. This property enables footsteps to be detected several meters from the sensing node but also introduces variability between buildings and sensor locations. Consequently, an algorithm trained in one environment may require calibration, adaptation, or additional validation before being transferred to a different structure.

Previous studies have exploited this vibration information for several human-monitoring tasks. Succi et al. investigated footstep detection and approximate tracking from floor vibrations [10]. Pan et al. used structural vibrations for occupant-traffic estimation and pedestrian identification [17,19]. Poston et al. investigated indoor footstep localization using structural-dynamics instrumentation and showed that the complex propagation of vibrations in buildings requires localization methods adapted to the structural medium [11]. Mirshekari et al. subsequently addressed occupant localization from footstep-induced structural vibrations using adaptive multilateration in heterogeneous floor structures [12]. Drira et al. evaluated sparse vibration sensing and machine-learning methods for occupant detection and recognition in buildings [7]. Yu et al. proposed a piezoelectric vibration-sensor system for pedestrian counting [9]. Other studies have demonstrated that floor-vibration signatures can support gait analysis and the extraction of health-related information [18,20]. More recently, Chakraborty et al. released a structural-vibration gait dataset, acquired from 100 participants and including different floor types, sensor distances, and walking conditions, using geophone-based measurements [13]. These works confirm that structural vibrations contain information beyond simple presence detection.

Distributed sensing configurations have also been used to estimate pedestrian position and trajectory. Li et al. combined measurements from multiple triaxial seismometers using angle and time-difference-of-arrival information to localize and track pedestrians [8]. Related localization approaches have exploited networks of structural-vibration measurements and adapted propagation models to estimate footstep position within buildings [11,12]. More recently, Appelle et al. combined geophone measurements with deep learning for footstep-based pedestrian localization in built environments, illustrating the increasing capability of vibration-based systems when spatial sensing and more computationally intensive models are available [21]. Such configurations provide richer spatial information and can reduce ambiguities that would be difficult to resolve from a single measurement point. However, they also increase the number of devices, synchronization requirements, calibration effort, communication overhead, installation cost, and maintenance complexity. These constraints are particularly relevant in industrial retrofitting, where sensing systems should preferably be installed without interrupting production or substantially modifying existing infrastructure.

Conversely, a single-sensor configuration reduces deployment complexity but must infer pedestrian movement from the components of the local structural response. A triaxial accelerometer directly measures acceleration along three orthogonal axes but does not directly report semantic information such as whether a person is approaching, leaving, or moving along a particular route. This information must be extracted from patterns embedded in the temporal evolution of the measured signals. Machine-learning methods can therefore enhance the functional capability of the transducer by converting low-level acceleration measurements into high-level pedestrian-flow information. In this sense, the combination of a MEMS accelerometer, embedded acquisition electronics, lightweight feature extraction, and data-driven classification constitutes an AI-enhanced sensing system.

Table 2 compares representative studies that use vibration or floor-response measurements for pedestrian, occupancy, and gait monitoring. The comparison distinguishes between path orientation, defined here as the dominant axis or geometrical orientation of a movement, and travel sense, defined as the positive or negative displacement along that orientation. This distinction is relevant because detecting a corridor or path is not equivalent to determining whether the pedestrian is entering or leaving the monitored area.

These two directional quantities are defined for approximately linear passages. Circular trajectories, which were also included in the original experimental protocol to increase geometric diversity, do not admit a unique global X/Y path orientation or a unique positive/negative travel sense. They are therefore retained only in the original movement-label analysis and are excluded from the functional directional evaluation described below.

As shown in Table 2, previous studies have demonstrated the suitability of structural vibrations for presence detection, counting, identification, gait assessment, and trajectory reconstruction. Nevertheless, approaches that provide detailed trajectory or directional information commonly rely on several spatially distributed sensors. A single-sensor configuration could reduce installation complexity, synchronization requirements, and communication overhead, but it remains unclear to what extent the local structural response measured at a single point retains information about pedestrian movement direction. This study therefore investigates whether footstep-induced structural vibrations measured with a single triaxial MEMS accelerometer contain sufficient discriminative information to characterize predefined pedestrian movement patterns, with particular emphasis on path orientation and travel sense.

To investigate this question, a custom sensing device based on an ADXL355 low-noise accelerometer (Analog Devices, Inc., Wilmington, MA, USA) and an ESP32 microcontroller (Espressif Systems, Shanghai, China) was used to acquire the floor-vibration response. The primary analysis was formulated at the footstep-event level so that complete physical footsteps rather than individual densely sampled observations constituted the classification and data-partitioning units. Compact time-domain descriptors were extracted from each event and evaluated using a Random Forest classifier [22]. The original sample-level analysis is retained only as a legacy within-sequence reference to document the initial methodology and illustrate the effect of temporal dependence in densely sampled feature vectors.

Under the primary leakage-aware event-level evaluation, classification of the complete A–K movement-label set was difficult. However, when the linear trajectories were analyzed according to the functional directional quantities defined above, individual footsteps retained measurable information about X/Y path orientation. The compact 12-dimensional event-level descriptor representation achieved a balanced accuracy of 71.52%. In contrast, reliable positive/negative travel-sense discrimination could not be demonstrated from isolated footsteps.

The original sample-level procedure yielded substantially higher performance (97.09% accuracy), but this result is retained only as a within-sequence reference because temporally adjacent observations contain strongly overlapping raw-sample information and therefore do not constitute independent evaluation units.

These findings indicate that a single floor-mounted triaxial MEMS accelerometer can provide useful path-orientation information without cameras or spatially distributed vibration-sensor networks. The reported results should nevertheless be interpreted as an initial proof of concept under one participant, one acquisition session, one sensor position, and one floor structure. Generalization across participants, sessions, structures, and realistic industrial disturbances remains to be evaluated.

The principal contributions of this work are as follows:

1. A single-sensor proof-of-concept is investigated for extracting coarse pedestrian path-orientation information from footstep-induced structural vibrations while explicitly distinguishing path orientation from travel sense.
2. The triaxial floor-vibration response is experimentally analyzed to identify motion-dependent patterns associated with predefined pedestrian trajectories.
3. A lightweight machine-learning pipeline based on time-domain signal representations and Random Forest classification is developed to transform floor-vibration measurements into directional pedestrian information.
4. A compact sensing platform combining an ADXL355 low-noise MEMS accelerometer and an ESP32 microcontroller is implemented, providing a hardware architecture compatible with future edge-processing and IIoT integration.
5. The sensing concept is evaluated primarily using leakage-aware event-level cross-validation, showing above-chance X/Y path-orientation discrimination from withheld footsteps under one controlled experimental condition; the original sample-level analysis is retained only as a legacy within-sequence reference.

The remainder of this paper is organized as follows. Section 2 describes the physical basis of footstep-induced vibrations, the system requirements, sensor selection, hardware platform, experimental protocol, feature-extraction procedure, and classification model. Section 3 presents the experimental results and classification metrics. Section 4 discusses the significance of the findings, their implications for industrial and smart-building applications, and the limitations of the current validation. Finally, Section 5 summarizes the main conclusions and identifies future research directions.

## 2. Materials and Methods

### 2.1. Methodological Overview

The proposed system was developed to investigate whether the structural vibrations generated by pedestrian footsteps contain sufficient information to infer both path orientation and travel sense using a single sensing node. The methodology comprised five stages: (i) definition of the sensing requirements, (ii) selection and implementation of the acquisition hardware, (iii) generation and labeling of a vibration dataset, (iv) extraction of lightweight temporal features, and (v) training and evaluation of a supervised classification model using the available labeled dataset.

**Figure 1:**
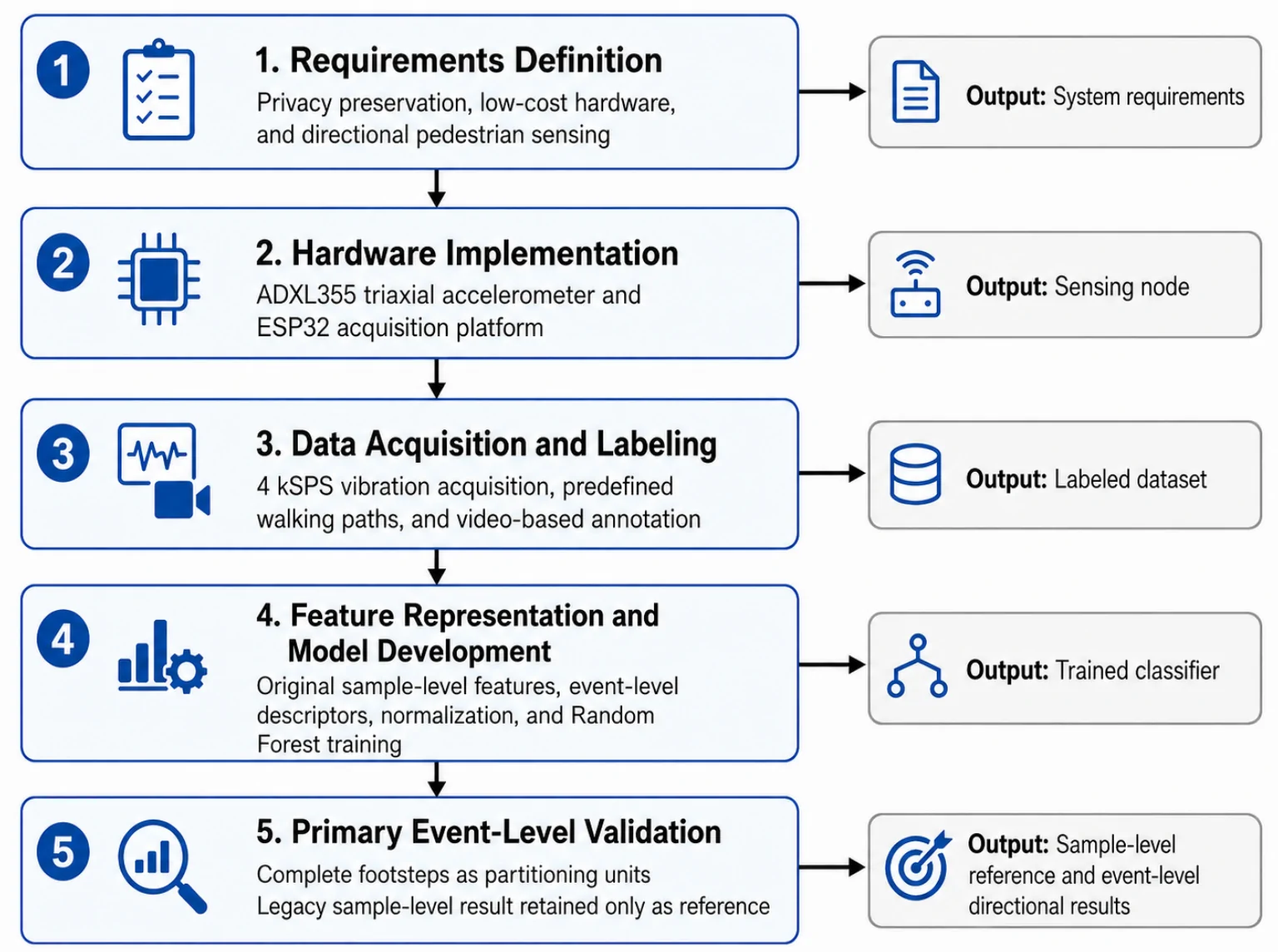
Methodological workflow of the proposed AI-enhanced directional pedestrian sensing system.

Figure 1 summarizes the complete workflow. A low-noise triaxial MEMS accelerometer was used to measure floor vibrations along the *x*-, *y*-, and *z*-axes. The acquired data were transmitted to a computer, converted into a structured dataset, synchronized with a video recording, and labeled according to the pedestrian movement performed around the sensor. The primary classification analysis was formulated at the footstep-event level. Complete labeled footsteps were represented using compact temporal descriptors and were partitioned exclusively between training and validation folds. The original sample-level representation and evaluation were retained only as a legacy reference for comparison with the initial implementation.

### 2.2. Physical Basis of Footstep-Induced Floor Vibrations

A pedestrian footstep applies a short-duration force to the floor and excites structural vibrations that propagate through the supporting structure. The measured response is a combination of the excitation generated during foot–floor contact and the dynamic characteristics of the floor, including its material, geometry, boundary conditions, and modal response [15,16].

Footstep-induced vibrations contain information over different frequency ranges. Low-frequency components are associated mainly with body motion and gait rhythm, whereas components at higher frequencies are related to the impulsive foot–floor interaction and to local structural resonances [7,23]. For the sensing task considered in this study, the principal frequency range of interest was defined between approximately 1 and 100 Hz. This interval contains the dominant components used for footstep detection and pedestrian-motion characterization while remaining compatible with low-complexity embedded acquisition and processing.

The vibration amplitudes and spectral content depend not only on the pedestrian motion but also on walking speed, footwear, distance from the sensor, and the properties of the supporting structure. The floor must therefore be regarded as part of the sensing system. This dependence motivates the use of supervised learning for the initial proof of concept and also limits the direct transferability of the trained model to floor structures that were not represented in the training data.

### 2.3. System Requirements

The sensing requirements were derived from the expected characteristics of walking-induced floor vibrations and from the constraints of an embedded measurement platform.

#### 2.3.1. Measurement Bandwidth

Relevant walking-induced floor vibrations are predominantly concentrated at low frequencies, with significant components typically found below 100 Hz [16]. The sensor and acquisition system were therefore required to provide an adequately characterized response over the 1–100 Hz range. This frequency interval was used as a reference design band for sensor sensitivity and noise considerations; it was not applied as a 1–100 Hz band-pass filter to the signals subsequently used for feature extraction.

#### 2.3.2. Noise Performance

Lee et al. reported walking-induced root-mean-square acceleration values on the order of 0.007 m/s^2^ in heavyweight buildings [16]. Expressed in units of gravitational acceleration, this value is

(1) $$a_{\mathrm{ref},\mathrm{rms}}=0.007\;m/s^{2}\frac{1\;g}{9.8\;m/s^{2}}\approx 0.714\;\mathrm{mg}.$$

This reported value was used as a reference amplitude for sensor selection, rather than as a universal minimum detectable footstep level. Assuming a design signal-to-noise ratio of 10 dB, a corresponding reference integrated noise level can be estimated as

(2) $$a_{\mathrm{noise},\mathrm{ref}}=\frac{a_{\mathrm{ref},\mathrm{rms}}}{10^{10/20}}\approx 226\;\mu g.$$

Considering an effective reference noise bandwidth of approximately 80 Hz, the corresponding target acceleration-noise density is

(3) $$ASD_{\mathrm{target}}=\frac{a_{\mathrm{noise},\mathrm{ref}}}{\sqrt{80\;\mathrm{Hz}}}\approx 25.3\;\mu g/\sqrt{\mathrm{Hz}}.$$

The resulting value was used only as a design criterion for comparing candidate accelerometers. An experimental characterization of the background acceleration of the complete installed sensing system is subsequently provided in Section 2.6 and Section 3.1. The assumed 10 dB signal-to-noise ratio is not an experimentally validated detection threshold, and the reported vibration amplitude from [16] should not be interpreted as a universal minimum footstep amplitude. Actual vibration levels depend on the pedestrian, sensor position, floor structure, and propagation conditions.

#### 2.3.3. Measurement Range and Resolution

Although the dynamic vibration amplitudes of interest are substantially below 1 g, the static acceleration caused by gravity must also be considered. Depending on the installation orientation, one measurement axis can contain a quasi-static component close to 1 g. A full-scale range of ±2 g was therefore selected to prevent saturation while maximizing sensitivity to low-amplitude floor vibrations.

To obtain an amplitude increment below 0.1 mg over a 4 g peak-to-peak range, the minimum required converter resolution is

(4) $$N_{\mathrm{bits}}\geq \log_{2}\left(\frac{4\;g}{0.1\;\mathrm{mg}}\right)\approx 15.3\;\mathrm{bits}.$$

A nominal digital resolution of at least 16 bits was therefore selected as a design requirement.

#### 2.3.4. Sampling Rate

A sampling rate higher than 200 samples per second would satisfy the Nyquist–Shannon criterion for a 100 Hz measurement bandwidth. Nevertheless, walking-induced vibrations contain impulsive and non-stationary components, and the preservation of their temporal shape requires a sampling rate substantially higher than the theoretical Nyquist limit. A minimum target sampling rate of 1 kSPS was therefore established during the system design. The experiments were ultimately conducted at 4 kSPS, corresponding to the maximum output data rate used with the selected accelerometer.

### 2.4. Vibration Sensor Selection

Several vibration-transducer technologies can be used to detect footstep-induced structural responses. Table 3 compares their principal characteristics from the perspective of embedded pedestrian-flow sensing.

The selection of the MEMS accelerometer should not be interpreted as a claim of superior low-frequency sensitivity over geophones. Geophones can provide high sensitivity to low-frequency structural vibrations, but the MEMS device was preferred here because it combines triaxial measurement, direct digital output, compact size, low-power operation, and straightforward integration with an embedded acquisition platform. In addition, the three orthogonal acceleration components provide a convenient basis for investigating whether the local structural response contains directional information.

The selected device was the ADXL355 low-noise, low-drift, triaxial MEMS accelerometer [24]. It provides 20-bit digital output, selectable measurement ranges, configurable internal filtering, and an SPI interface. The ±2 g range was used in this study, resulting in a nominal scale factor of approximately 3.9 μg/LSB. The datasheet specifies a noise density of approximately $25\;\mu g/\sqrt{\mathrm{Hz}}$ for the selected measurement range [24]. This value is consistent with the target noise-density criterion derived in Equation (3).

### 2.5. Embedded Acquisition Platform

The acquisition platform consisted of an ADXL355 accelerometer (Analog Devices, Inc., Cavite, Philippines) and an ESP32-WROOM-32D microcontroller (Espressif Systems, Shanghai, China) module mounted on a custom printed circuit board. The ESP32 integrates a dual-core Xtensa LX6 processor operating at up to 240 MHz, 520 kB of SRAM, SPI and UART interfaces, and Wi-Fi and Bluetooth Low Energy connectivity [25]. The SPI peripheral was used to communicate with the accelerometer.

**Figure 2:**
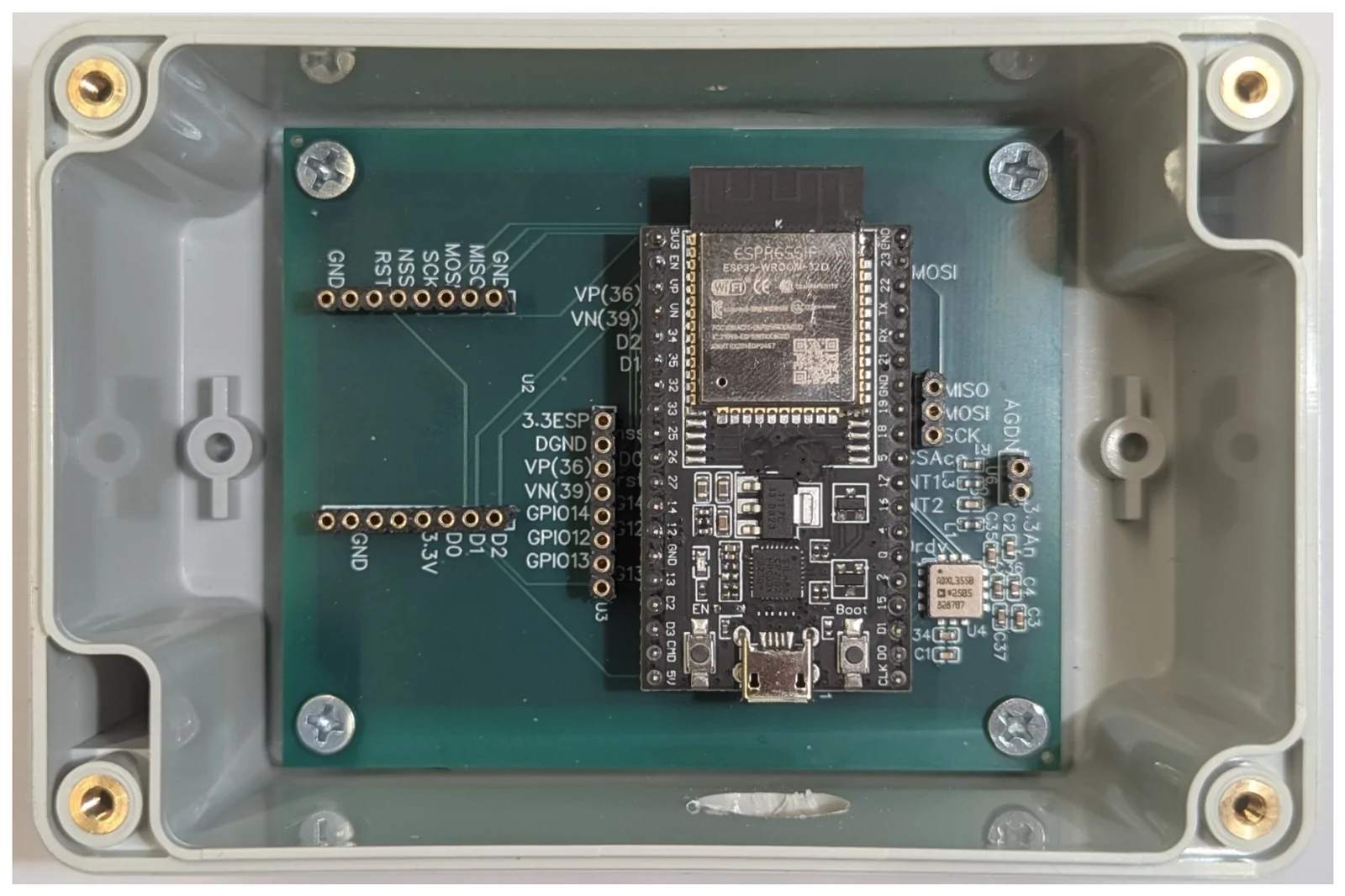
Custom acquisition platform integrating the ADXL355 triaxial MEMS accelerometer and the ESP32 microcontroller.

The custom PCB was designed to minimize wiring length and avoid the mechanical and electrical instability associated with temporary prototyping connections. The board also included an interface reserved for an optional LoRa communication module. For the experiments reported in this study, the device was enclosed in a protective case and placed directly on the floor, as shown in Figure 2.

The ESP32 acquired the digital acceleration data from the ADXL355 through the SPI interface and transmitted them to a laptop through its UART-to-USB connection. The ESP32 performed sensor configuration and continuous data acquisition, whereas the computer was used for data recording, decoding, labeling, and offline machine-learning analysis.

At the selected output data rate, transmitting each sample as formatted decimal text would have exceeded the available serial throughput. Therefore, each triaxial sample was transmitted in binary form as three signed 32-bit integer values without textual separators, resulting in a payload of 12 bytes per sample.

With standard 8N1 asynchronous transmission, each transmitted byte requires 10 bits. Consequently, one triaxial sample requires

(5) $$N_{\mathrm{UART}}=12\;\mathrm{bytes}\times 10\;\mathrm{bits}/\mathrm{byte}=120\;\mathrm{bits}.$$

At a UART transmission rate of 1 Mbit/s, the corresponding theoretical transmission time is

(6) $$t_{\mathrm{UART}}=\frac{120\;\mathrm{bits}}{10^{6}\;\mathrm{bits}/s}=120\;\;\mu s.$$

This value is lower than the 250  μs sampling interval corresponding to a sampling rate of 4 kSPS, allowing the acquired samples to be transmitted continuously under the experimental conditions.

**Figure 3:**
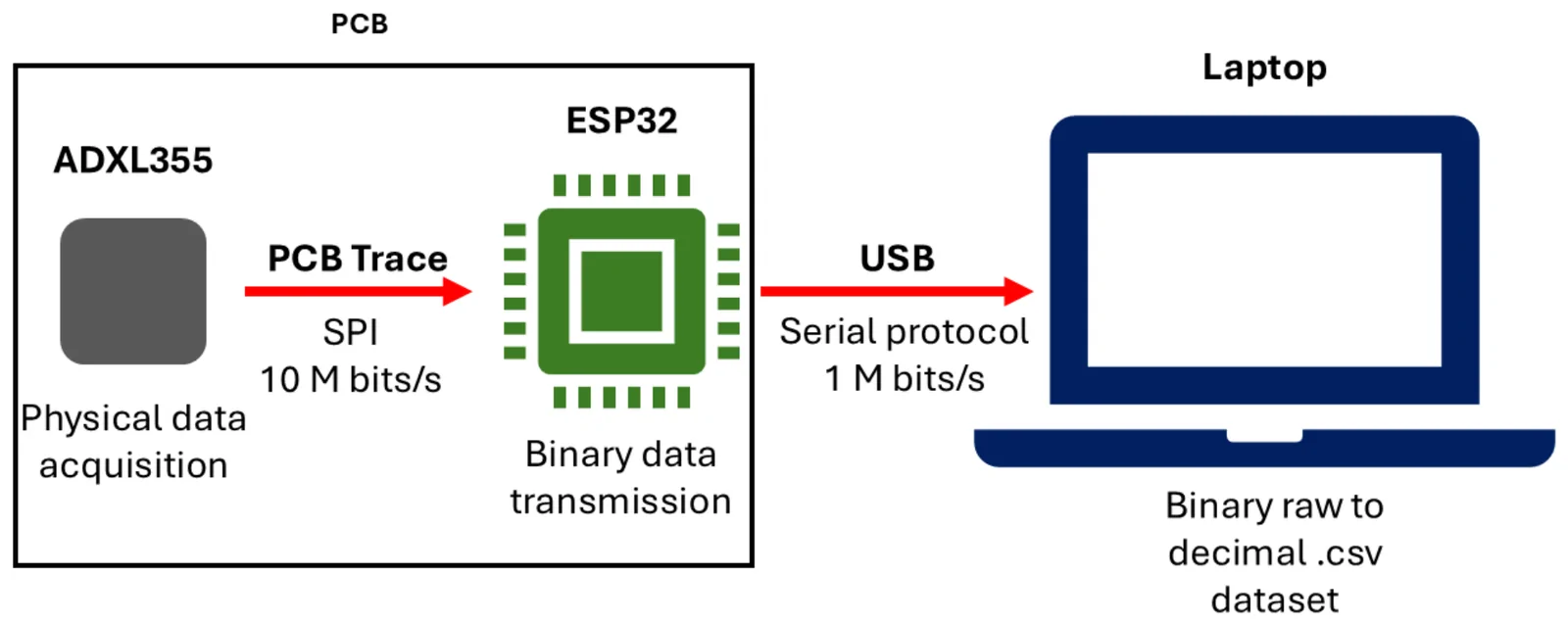
Experimental setup used for floor-vibration acquisition.

Figure 3 summarizes the complete acquisition chain. The binary stream received by the computer was recorded and subsequently decoded into a CSV file containing the *x*-, *y*-, and *z*-axis acceleration values.

It should be noted that the ESP32 was used only for sensor configuration, data acquisition, and transmission in the present study. Feature extraction and Random Forest inference were performed offline on the computer. The proposed platform is therefore compatible with future edge implementation, but the present experiments do not yet constitute a complete on-device AI deployment.

### 2.6. Experimental Environment and Data Acquisition

The experimental campaign was conducted in a controlled indoor room of approximately 5 m × 3 m, located one story above ground level. The floor was therefore a suspended structure over an occupied space, rather than a slab resting on the ground. It consisted of steel joists of approximately 0.5 m spacing with ceramic infill elements spanning between them, covered by a leveling fill and finished with polished in situ terrazzo. Because the low-frequency structural response is governed by the supporting structure rather than by the surface finish, both are reported here to enable comparison with other test structures. The room was unoccupied except for the participant, and the acquisition was performed outside working hours to minimize ambient vibration from adjacent activity.

The sensor enclosure was positioned on the floor near the center of the measurement area and remained fixed throughout the acquisition. It rested freely on the floor surface under its own weight, without adhesive or mechanical fixing. The device orientation defined the positive *x*- and *y*-axes used to describe the pedestrian trajectories, whereas the *z*-axis was approximately normal to the floor surface.

The acceleration signals were acquired at 4 kSPS, the selected maximum output data rate of the ADXL355. At this setting, the internal low-pass filter was configured with a cutoff frequency of approximately 1 kHz. This bandwidth is intentionally wider than the 1–100 Hz reference band used during sensor selection. The latter was employed only for sensitivity and noise-design considerations and was not imposed as an analysis filter. The wider acquisition bandwidth preserves the transient waveform and allows the recorded dataset to support subsequent analyses involving higher-frequency signal components. The influence of this bandwidth difference on the experimentally measured background level is quantified in Section 3.1.

The internal high-pass filter of the ADXL355 was enabled with a cutoff frequency of 0.155 Hz to attenuate the quasi-static gravity component and slow baseline variations while preserving the dynamic vibration response. The total acquisition duration was 90 s. At 4 kSPS, this produced 360,000 samples for each acceleration axis.

A single adult participant (75 kg body mass, 1.73 m height) performed the predefined movements in the vicinity of the sensor, wearing flat rubber-soled shoes. The participant walked at a self-selected comfortable pace, corresponding to an approximate cadence of 100 steps per minute as estimated from the synchronized video recording. No instructions were given regarding step length or foot placement beyond following the predefined paths.

The protocol included linear trajectories parallel to both horizontal axes. Additional circular trajectories of approximately 0.3 m and 1.5 m radius, concentric with the sensor position, were included to introduce non-linear movement patterns. These circular movements were included to increase the diversity of the original movement-label experiment. Because a complete circular passage does not have a unique global X/Y path orientation or a unique positive/negative travel sense, the circular classes were not used in the subsequent functional directional analysis. Each predefined trajectory was executed once within a single continuous 90 s recording. Intervals not corresponding to one of the predefined trajectories, including transitions between movements, were assigned to an indeterminate class. Samples without pedestrian-step activity were assigned to a non-step class.

**Figure 4:**
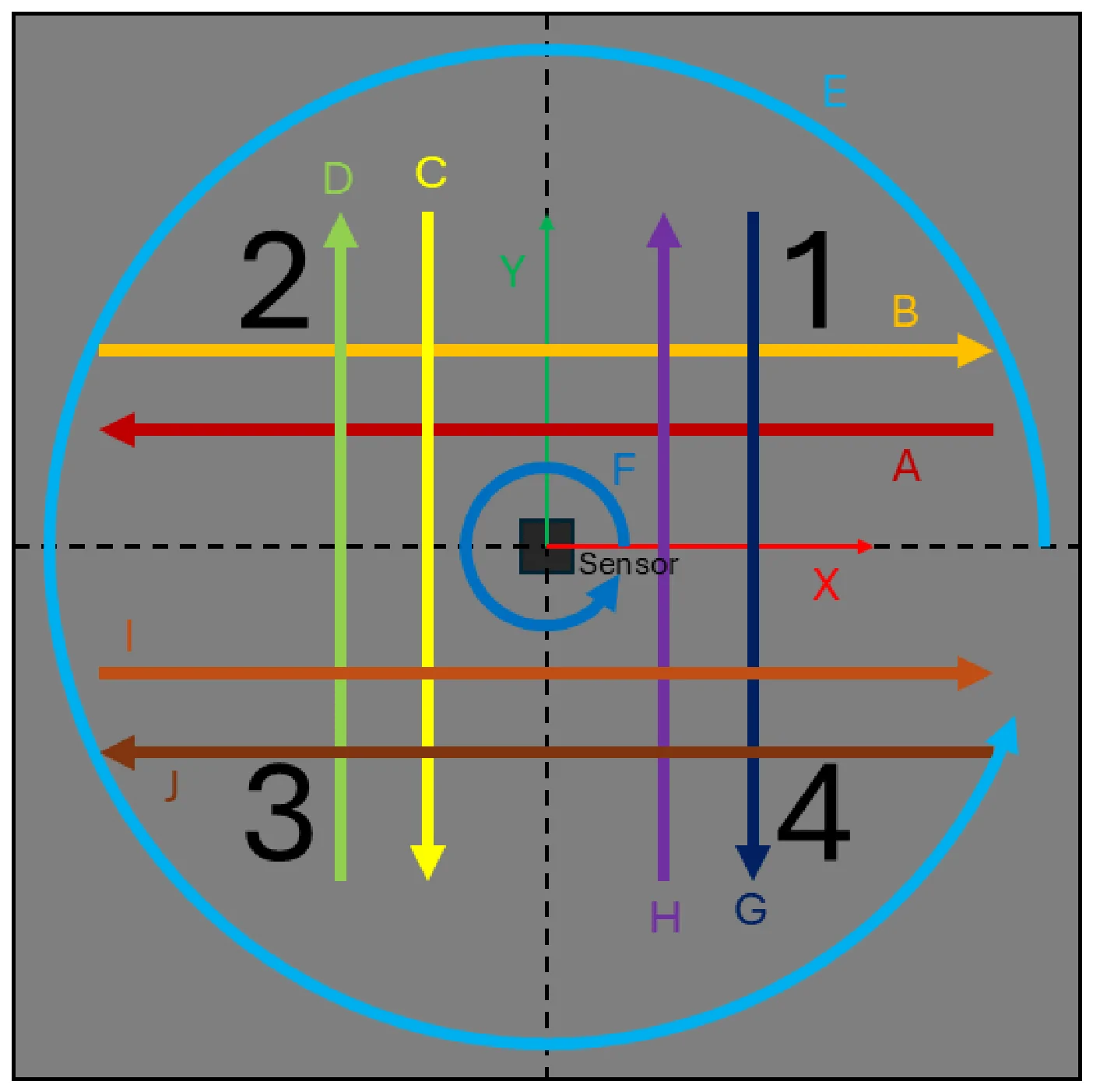
Definition of the pedestrian movement classes relative to the sensor coordinate system.

Figure 4 illustrates the movement classes and their orientation relative to the sensing node.

The experiment was recorded on video synchronized with the acceleration measurements by means of an initial impact event visible in both the video and the vertical acceleration channel. The video was used to assign each labeled interval to the movement being performed and to identify the corresponding footstep events. The complete labeled dataset generated in this study, together with the video recording used as the labeling reference, is openly available in Zenodo [26]. The labeling process generated the following classes:

- linear movements along the positive and negative *x*-directions;
- linear movements along the positive and negative *y*-directions;
- small and large circular movements;
- indeterminate or transitional movements; and
- non-step intervals.

The complete A–K label set was retained as a legacy movement-label classification problem corresponding to the original experimental design. For the primary directional analysis, only the linear trajectory classes were subsequently regrouped according to two explicitly defined functional quantities: X/Y path orientation and positive/negative travel sense. Circular and heterogeneous labels were excluded from this functional analysis because they do not correspond to a unique linear orientation and signed displacement. In a practical industrial deployment, these geometric labels could be mapped to functional classes such as entry, exit, longitudinal passage, transverse passage, or unknown movement.

#### Experimental Background-Noise Characterization

To complement the design-stage noise analysis of Section 2.3.2, the background acceleration of the installed sensing system was characterized directly from the experimental recording. This analysis was performed on the original triaxial acceleration signals expressed in units of g, before applying the feature-extraction procedures described in Section 2.7.

Background intervals were selected objectively from the gaps between consecutive manually annotated footstep events. A candidate interval was considered valid only when a 0.5 s segment could be placed between two consecutive footsteps while maintaining a minimum temporal separation of 250 ms from the nearest annotated footstep on either side. Using this criterion, seven non-overlapping 0.5 s background intervals distributed throughout the acquisition were obtained. Each interval therefore contained 2000 samples per acceleration axis at the 4 kSPS acquisition rate.

For each interval and acceleration component, the mean value was removed and the power spectral density (PSD) was estimated using Welch’s method with a 2000-sample Hann window and no overlap. Since each interval contained 2000 samples at 4 kSPS, this resulted in a frequency resolution of 2 Hz. The corresponding amplitude spectral density (ASD) was obtained as the square root of the PSD. Band-limited RMS background acceleration was then calculated by integrating the PSD over the 1–80 Hz range,

(7) $$a_{\mathrm{RMS},1-80}=\sqrt{\int_{1}^{80}S_{a}\left(f\right)\;df},$$

where $S_{a}\left(f\right)$ denotes the acceleration PSD. The 1–80 Hz interval was used for this band-limited calculation to enable direct comparison with the approximately 80 Hz effective noise bandwidth adopted in the design-stage analysis of Section 2.3.2; the broader 1–100 Hz interval remains the reference frequency range of interest for footstep sensing.

Broadband RMS was calculated directly from each mean-removed 0.5 s acceleration segment using the complete recorded waveform. Consequently, this value includes the substantially wider acquisition bandwidth shaped by the approximately 1 kHz internal low-pass filter.

**Figure 5:**
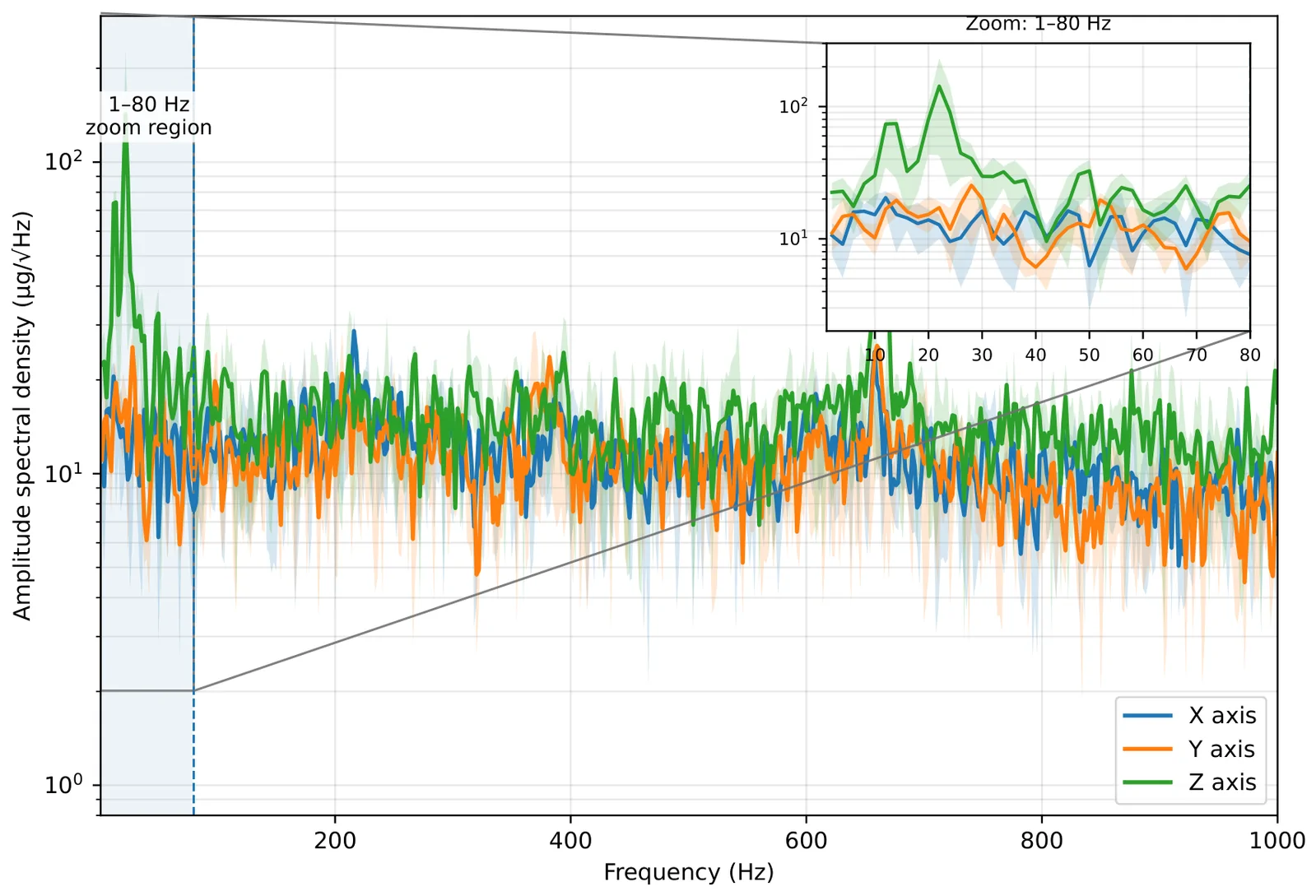
Experimental background acceleration amplitude spectral density estimated from seven 0.5 s inter-step intervals, each located at least 250 ms from the nearest annotated footstep. Solid curves show the mean ASD for the X-, Y-, and Z-components, and shaded regions indicate the interquartile range. The main plot shows the spectrum up to 1 kHz, while the inset enlarges the 1–80 Hz region. The vertical dashed line marks the 80 Hz effective reference bandwidth used in the preliminary sensor-selection noise analysis.

For each background interval, the 1–80 Hz RMS acceleration was computed by integrating its individual PSD. The reported RMS values therefore correspond to the mean and standard deviation of the seven interval-wise RMS estimates. Separately, the spectrum shown in Figure 5 was obtained by averaging the ASD values across the seven intervals at each frequency.

This procedure characterizes the background acceleration measured by the complete installed sensing system rather than the intrinsic self-noise of the ADXL355 alone. The measured background therefore includes contributions from the accelerometer, acquisition electronics, sensor–floor coupling, supporting structure, residual environmental vibration, and any residual structural response persisting between annotated footstep events.

### 2.7. Signal Representation and Feature Extraction

The primary event-level analysis was based on footstep-centered representations constructed from complete physical footsteps. The compact 12-dimensional descriptor vector $d_{e}$ was used as the principal low-dimensional representation. For methodological continuity with the original sample-level feature design, a higher-dimensional temporally ordered representation $z_{e}$ was also constructed from the original instantaneous and moving-average features, and the concatenated representation $u_{e}$ was evaluated as an additional comparison.

#### 2.7.1. Sample-Level Feature Representation

Let

(8) $$a\left[n\right]={\left[a_{x}\left[n\right]\;a_{y}\left[n\right]\;a_{z}\left[n\right]\right]}^{T}$$

denote the triaxial acceleration sample acquired at time index *n*. In addition to the instantaneous acceleration components, moving averages were computed to represent the recent temporal evolution of the vibration signals.

For an axis q∈{x,y,z} and a window containing *M* samples, the moving-average feature was calculated as

(9) $${\bar{a}}_{q,M}\left[n\right]=\frac{1}{M}\sum_{k=0}^{M-1}a_{q}\left[n-k\right].$$

Four window lengths were retained from the original implementation:

(10) M∈{25,50,100,250}.

At a sampling frequency of 4 kSPS, these windows correspond to durations of 6.25, 12.5, 25, and 62.5 ms, respectively. The different temporal scales were selected to represent both short variations around the footstep impact and the slower evolution of the damped structural response.

The sample-level feature vector was therefore defined as

(11) $$x\left[n\right]={\left[a_{x}\left[n\right],a_{y}\left[n\right],a_{z}\left[n\right],{\bar{a}}_{x,25}\left[n\right],{\bar{a}}_{y,25}\left[n\right],{\bar{a}}_{z,25}\left[n\right],\ldots ,{\bar{a}}_{x,250}\left[n\right],{\bar{a}}_{y,250}\left[n\right],{\bar{a}}_{z,250}\left[n\right]\right]}^{T}.$$

The resulting representation contains 15 features: the 3 instantaneous acceleration components and 12 moving-average features. This representation was used in the original sample-level reference evaluation described in Section 2.9.

#### 2.7.2. Footstep-Centered Event Representation

The original sample-level representation produces one feature vector for every acquired sample. At 4 kSPS, consecutive feature vectors are separated by only 0.25 ms, while the longest moving-average feature contains 250 samples (62.5 ms). Consequently, consecutive observations contain strongly overlapping information. To enable the primary event-level evaluation, in which complete physical events rather than individual samples constitute the classification units, a footstep-centered representation was constructed.

The dataset contains 118 manually identified footsteps. For each footstep *e*, the sample corresponding to the most significant vibration peak, denoted by $p_{e}$, was associated with its corresponding movement class using the synchronized video annotation. Since the onset and end of each individual footstep response were not manually annotated, a fixed local vibration window was defined around each labeled peak rather than attempting to estimate exact event boundaries.

A window containing 1000 samples (250 ms) was extracted for each event, corresponding approximately to 125 ms before and 125 ms after the labeled peak:

$$W_{e}=\left\{p_{e}-500,\ldots ,p_{e}+499\right\}.$$

The 250 ms duration was selected to retain the local pre-impact and post-impact vibration evolution while avoiding overlap between the event windows in the available acquisition. The minimum observed separation between two consecutive labeled footstep peaks was 1510 samples (377.5 ms), which is larger than the 1000-sample event window.

Each event window was subsequently divided into ten consecutive and non-overlapping temporal intervals containing 100 samples each:

$$W_{e}=\left\{I_{e,1},I_{e,2},\ldots ,I_{e,10}\right\},$$

where each interval represents 25 ms of the vibration response.

For every interval, the mean value of each of the 15 original sample-level features was calculated:

$$v_{e,r}=\frac{1}{100}\sum_{n\in I_{e,r}}x\left[n\right],\;r=1,\ldots ,10.$$

Thus, each $v_{e,r}$ contains the same 15 physical and temporal descriptors used in the original method. Averaging was performed only within each 25 ms interval to reduce sensitivity to individual sample fluctuations. The ten intervals were not averaged together.

Instead, their temporal order was explicitly preserved by concatenating the ten interval-level vectors:

$$z_{e}={\left[v_{e,1}^{T},v_{e,2}^{T},\ldots ,v_{e,10}^{T}\right]}^{T}.$$

Each footstep was therefore represented by a single 150-dimensional event vector,

$$z_{e}\in R^{150},$$

obtained exclusively from the 15 features already defined in the original methodology. The increased dimensionality does not correspond to the introduction of additional physical descriptors; instead, it preserves the temporal evolution of the original features across ten successive regions of the footstep response. The ten intervals are non-overlapping in terms of their sample indices. However, because some components of x[n] are causal moving averages, adjacent interval summaries can share raw-sample support within the same footstep. This within-event dependence does not generate information leakage across cross-validation folds because the complete footstep representation is assigned exclusively to a single fold and the raw-sample supports of different footstep events do not overlap.

Because this representation is high-dimensional relative to the limited number of labeled events, it was included only as a continuity comparison with the original feature design and is not proposed as the preferred event-level representation. The compact descriptor vector introduced below provides a substantially lower-dimensional alternative.

#### 2.7.3. Event-Level Temporal Descriptors

In addition to preserving the temporal evolution of the original 15 features, a compact set of conventional time-domain descriptors was extracted directly from each 250 ms footstep window. This complementary representation was introduced to assess whether descriptors that characterize the complete vibration event provide more stable information than the moving-average features originally defined at the sample level.

For each acceleration component q∈{x,y,z}, four descriptors were calculated over the N=1000 samples of the corresponding footstep window. The root-mean-square acceleration was defined as

(12) $$a_{q,\mathrm{RMS}}=\sqrt{\frac{1}{N}\sum_{n\in W_{e}}a_{q}^{2}\left[n\right]}.$$

The maximum absolute acceleration was calculated as

(13) $$a_{q,\max}=\max_{n\in W_{e}}\left|a_{q}\left[n\right]\right|,$$

whereas the peak-to-peak amplitude was obtained as

(14) $$a_{q,P2P}=\max_{n\in W_{e}}a_{q}\left[n\right]-\min_{n\in W_{e}}a_{q}\left[n\right].$$

Finally, the kurtosis of each acceleration component was included to characterize the impulsiveness of the vibration event:

(15) $$\kappa_{q}=\frac{\frac{1}{N}\sum_{n\in W_{e}}{\left(a_{q}\left[n\right]-\mu_{q}\right)}^{4}}{{\left[\frac{1}{N}\sum_{n\in W_{e}}{\left(a_{q}\left[n\right]-\mu_{q}\right)}^{2}\right]}^{2}},$$

where $\mu_{q}$ denotes the mean acceleration within the event window.

The resulting event-level temporal descriptor vector was therefore

(16) $$d_{e}={\left[a_{x,\mathrm{RMS}},a_{y,\mathrm{RMS}},a_{z,\mathrm{RMS}},a_{x,\max},a_{y,\max},a_{z,\max},a_{x,P2P},a_{y,P2P},a_{z,P2P},\kappa_{x},\kappa_{y},\kappa_{z}\right]}^{T},$$

yielding 12 additional descriptors per footstep.

These descriptors were selected because they characterize complementary properties of the complete vibration event while retaining low computational complexity. RMS acceleration represents the overall vibration magnitude, maximum and peak-to-peak amplitudes characterize the event amplitude range, and kurtosis provides information on the impulsive nature of the footstep-induced response.

Three feature configurations were considered in the event-level analysis: (i) the temporally ordered representation derived exclusively from the 15 original features, $z_{e}$; (ii) the 12 event-level temporal descriptors, $d_{e}$; and (iii) their concatenation,

(17) $$u_{e}={\left[z_{e}^{T}\;d_{e}^{T}\right]}^{T},$$

resulting in a 162-dimensional combined representation. All three configurations were evaluated using exactly the same leakage-aware cross-validation procedure described in Section 2.9.

**Figure 6:**
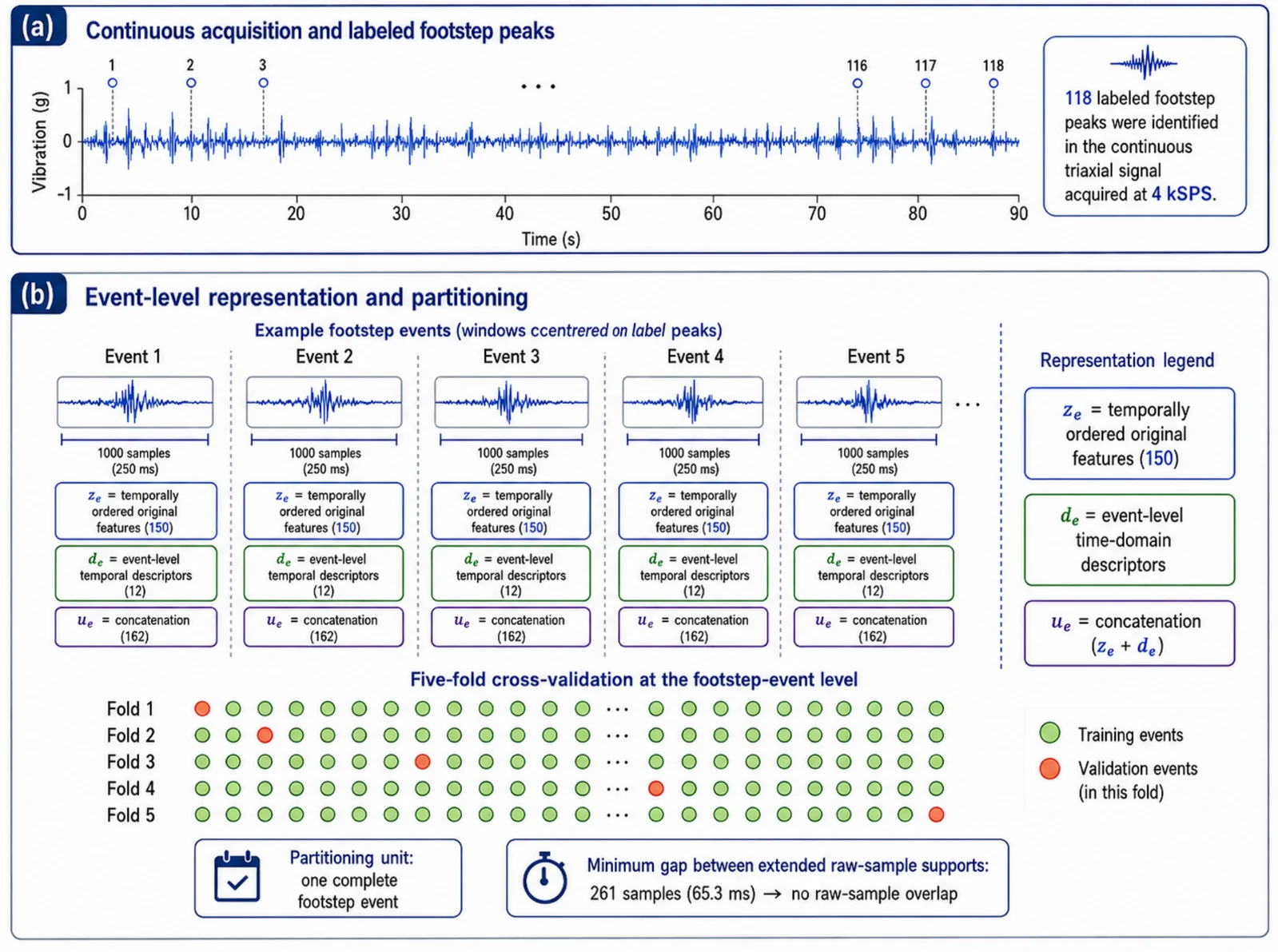
Event-level representation and leakage-aware validation. (**a**) Labeled footstep peaks in the continuous triaxial vibration signal. (**b**) Construction of footstep-centered representations and five-fold event-level cross-validation, with complete footsteps assigned exclusively to training or validation.

Figure 6 summarizes the event-level processing strategy. Panel (a) illustrates the identification of labeled footstep peaks in the continuous vibration recording, whereas panel (b) shows the construction of the footstep-centered representations and the event-level partitioning used for cross-validation. The original 15 lightweight features are preserved, while event-level grouping avoids sample-level leakage and retains the temporal evolution within each footstep.

Each event vector inherited the movement label associated with the corresponding annotated footstep peak. Therefore, all information derived from one physical footstep constituted a single classification observation in the event-level analysis.

The use of the causal 250-sample moving average was also taken into account when assessing raw-sample overlap between consecutive event representations. Although each event window contains 1000 samples, the first feature vector within the window can depend on the preceding 249 raw samples. Consequently, the complete raw-sample support associated with an event extends from approximately

$$p_{e}-749\;\mathrm{to}\;p_{e}+499.$$

Given the minimum observed peak-to-peak separation of 1510 samples, the raw-sample supports of the two closest events remain separated by 261 unused samples, corresponding to approximately 65.3 ms. This separation exceeds the 250-sample exclusion criterion associated with the longest moving-average window and ensures that no raw acceleration sample contributes simultaneously to the feature representation of two consecutive footsteps.

### 2.8. Classification Models

A Random Forest classifier was used to map the feature vectors to the predefined movement classes. Random Forest is an ensemble-learning method in which multiple decision trees are trained using bootstrapped subsets of the training data and randomized subsets of features [22]. For classification, the final predicted class is obtained by majority voting among the individual trees. Each tree retains an explicit sequence of feature-based decisions from the root node to the predicted class, providing a degree of interpretability that is generally not available in more complex deep-learning models.

The method was selected because it can represent non-linear decision boundaries, supports multiclass classification, is comparatively robust to feature scaling and outliers, and has a relatively low inference cost. Random Forest inference is based mainly on threshold comparisons and decision-tree traversal and can therefore be implemented on resource-constrained embedded platforms. Its actual memory footprint and execution cost, however, depend on the number and depth of the retained trees. The tree-based structure also provides greater interpretability than many deep-learning models, because individual decision paths and feature-importance measures can be inspected. These properties are consistent with the long-term objective of implementing the classifier directly on the sensing node, although classifier inference was performed offline in the present study.

The model was trained using the labeled feature representations described in Section 2.7. The Random Forest configuration was inherited from the original implementation and was kept fixed in the revised event-level analyses to avoid additional model selection on the limited event-level dataset.

The original sample-level analysis was implemented using Scikit-learn (version 0.22), whereas the revised event-level analyses reported in this manuscript were reproduced using Scikit-learn (version 1.8.0). In both cases, the classifier configuration corresponded to an ensemble of 100 decision trees (n_estimators=100) with no explicit maximum tree depth (max_depth=None). The Gini impurity criterion was used (criterion=’gini’), and the number of candidate features considered at each split corresponded to the square root of the total number of input features. In the revised implementation, this was explicitly specified as max_features=’sqrt’. Bootstrap sampling was enabled (bootstrap=True), nodes required at least two samples to be split (min_samples_split=2), and terminal leaves contained at least one sample (min_samples_leaf=1). No class weighting was applied (class_weight=None), and a fixed random seed (random_state=42) was used for reproducibility.

The same classifier configuration was used for all revised event-level feature representations and ablation analyses. No additional hyperparameter tuning was performed after defining the leakage-aware validation strategy.

To assess whether the Random Forest remained an appropriate choice for the event-level directional classification task, four additional low-complexity classifiers were evaluated: logistic regression, a linear support vector machine (Linear SVM), linear discriminant analysis (LDA), and a compact multilayer perceptron (MLP). These alternatives were selected to represent complementary classifier families while retaining compatibility with future resource-constrained embedded implementation. Logistic regression provides a simple linear probabilistic baseline, Linear SVM provides a margin-based linear classifier, LDA provides a low-complexity discriminant classifier, and the MLP introduces non-linear decision boundaries using a compact neural-network architecture.

The classifier comparison was performed using the 12-dimensional event-level temporal descriptor representation $d_{e}$, since this representation provided the best X/Y path-orientation performance in the leakage-aware analysis while maintaining a low feature dimensionality. Logistic regression was implemented using L2 regularization with C=1, the lbfgs solver, an intercept term, no class weighting, and a maximum of 2000 optimization iterations.

Linear SVM was implemented using a linear support-vector classifier with C=1, no class weighting, a maximum of 5000 optimization iterations, and random_state=42.

LDA was implemented using the default singular-value-decomposition solver (solver=’svd’), without shrinkage, with class priors estimated from the training data. The compact MLP contained one hidden layer with eight neurons, yielding a 12–8–2 architecture for the binary path-orientation task. ReLU activation and the Adam optimizer were used, with an L2 regularization coefficient of $\alpha =10^{-4}$, an initial learning rate of $10^{-3}$, and a maximum of 2000 training iterations. Early stopping was not enabled. A fixed random seed of 42 was used for the Random Forest, Linear SVM, and MLP implementations.

No classifier-specific hyperparameter search was performed. Accordingly, the classifier comparison should be interpreted as a fixed-model baseline comparison rather than as a comprehensive bias–variance optimization study. With only 59 linear footstep events available for the primary path-orientation task, extensive hyperparameter optimization or nested model-selection procedures would provide highly uncertain estimates and could introduce additional selection bias. A rigorous bias–variance analysis therefore requires a larger dataset with independent experimental groups. To isolate the effect of classifier choice, all five models were evaluated using exactly the same five stratified event-level cross-validation partitions (shuffle=True, random_state=42), the same 59 labeled linear footstep events, and the same training-only min–max normalization procedure. Accuracy, balanced accuracy, and macro-F1 were calculated independently for each validation fold as described in Section 2.10.

### 2.9. Training and Validation Strategy

The primary evaluation strategy was a leakage-aware grouped cross-validation procedure in which complete footstep events constituted the data-partitioning units. This procedure was used for all principal event-level results reported in this manuscript. The original sample-level evaluation from the initial implementation is retained separately as a legacy within-sequence reference; it is not used as evidence of generalization.

A fixed random seed (random_state=42) was used where applicable throughout the model-development and evaluation procedures to ensure reproducibility. Unless otherwise stated, the Random Forest configuration described in Section 2.8 was kept unchanged. In particular, the leakage-aware evaluation was performed using the same classifier configuration and was not used to retune the model hyperparameters.

#### 2.9.1. Leakage-Aware Event-Level Evaluation

As illustrated in Figure 6b, the revised evaluation uses complete physical footsteps as the partitioning units. All information derived from the same footstep is assigned exclusively to either the training or the validation subset. Each footstep is represented by the event-level feature representations defined in Section 2.7.

The released dataset contains 118 manually identified footsteps. For each event, the sample corresponding to the most significant vibration peak was associated with its corresponding movement label using the synchronized video recording. These labeled footsteps constitute the withheld footstep events, used in the event-level analysis.

A five-fold stratified cross-validation procedure was applied at the footstep-event level. The folds were constructed to preserve the movement-class distribution as far as permitted by the number of available events in each class. In each iteration, complete footstep events were withheld for validation, and the remaining events were used for training. Therefore, every validation prediction corresponded to a physical footstep whose event representation had contributed no information to the corresponding training fold.

Absence of raw-sample overlap between adjacent event representations was verified by considering the raw-sample support of the longest moving-average feature. The closest pair of labeled footstep peaks in the dataset was separated by 1510 samples. After accounting for the 1000-sample event window and the preceding 249 samples required by the causal M=250 moving average, the raw-sample supports of the two closest events remained separated by 261 unused samples, corresponding to approximately 65.3 ms. Since this separation exceeds the 250-sample (62.5 ms) support of the longest moving-average feature, no raw acceleration sample contributes simultaneously to two consecutive event representations.

Preprocessing was performed independently within every cross-validation fold. Let $f_{j}$ denote the *j*th component of the feature representation considered in a given experiment, corresponding to either the temporally ordered representation $z_{e}$, the event-level descriptor vector $d_{e}$, or the combined representation $u_{e}$. For each feature dimension, the minimum and maximum values were estimated exclusively from the footsteps assigned to the training folds,

(18) $$f_{j}^{\prime }=\frac{f_{j}-f_{j,\min}^{\mathrm{train}}}{f_{j,\max}^{\mathrm{train}}-f_{j,\min}^{\mathrm{train}}},$$

and the resulting normalization parameters were subsequently applied unchanged to the footsteps in the validation fold. Validation events therefore contributed no information to feature normalization, model fitting, or parameter estimation. Consequently, a separate set of normalization parameters was obtained for each cross-validation fold, and no statistics computed from the corresponding validation events were used during preprocessing.

Because only 118 labeled footstep events were available, no additional event-level hold-out subset was separated from the grouped cross-validation data. Further partitioning would have substantially reduced the number of available training and validation events, particularly for the least represented movement classes. Leakage-aware performance is therefore reported as the mean and standard deviation across the five folds using accuracy, balanced accuracy, and macro-averaged F1-score.

The event-level evaluation provides a substantially more conservative assessment than the original sample-level partition because the statistical unit used for classification is now a complete physical footstep rather than an individual sample from a densely sampled continuous signal. Training and validation therefore contain different footstep events, and neither event representations nor the raw acceleration samples contributing to them are shared between the two subsets.

Nevertheless, event-level separation between training and validation should not be interpreted as independence at the trajectory, session, participant, or structural level. All footsteps were acquired from the same participant, during the same acquisition session, at the same sensor position, and on the same floor structure. The present dataset therefore does not permit participant-wise, session-wise, or cross-structure validation. Accordingly, the leakage-aware results quantify generalization to unseen footstep events within the experimental conditions considered in this study.

#### 2.9.2. Original Sample-Level Evaluation

In the original evaluation, the complete labeled sample-level dataset was randomly divided into a development subset containing 80% of the available samples and a sample-level hold-out subset containing the remaining 20%. Five-fold cross-validation was subsequently applied to the 80% development subset. In each iteration, four folds were used for model training and the remaining fold for validation, corresponding to approximately 64% and 16% of the complete sample-level dataset, respectively.

After model development, a new Random Forest classifier with the selected configuration was trained using the complete 80% development subset and evaluated on the remaining 20% sample-level hold-out subset.

For every training/validation partition, the min–max normalization parameters were estimated exclusively from the corresponding training samples. The resulting minimum and maximum values were then applied unchanged to the associated validation data. Likewise, for the final sample-level evaluation, the normalization parameters were estimated exclusively from the complete development subset and subsequently applied to the hold-out subset.

This original procedure provides a measure of sample-level separability within the recorded acquisition sequence. However, it does not ensure temporal independence between the training and validation observations. Feature vectors are generated at the 4 kSPS acquisition rate, whereas the longest moving-average feature contains $M_{\max}=250$ samples, corresponding to 62.5 ms. Consequently, two feature vectors associated with consecutive samples can share up to 249 of the 250 raw acceleration samples used to compute the longest moving average.

Because the original folds and hold-out subset were generated at the sample level, temporally adjacent observations and strongly overlapping feature windows could therefore be assigned to different subsets. The performance obtained using this procedure is consequently retained in the revised manuscript as a within-sequence sample-level reference and should not be interpreted as an estimate of generalization to temporally withheld footstep events, complete trajectories, acquisition sessions, participants, or floor structures.

### 2.10. Evaluation Metrics

Classification performance was evaluated using the confusion matrix, overall accuracy, macro-averaged F1-score, and balanced accuracy. For the primary leakage-aware event-level evaluation, these metrics were calculated independently within each of the five footstep-level validation folds and are reported as mean and standard deviation across folds. For the legacy sample-level reference, the same metrics were calculated once on the original 20% sample-level hold-out subset.

Overall accuracy was calculated as the proportion of correctly classified observations over the total number of observations in the corresponding evaluation subset:

(19) $$\mathrm{Accuracy}=\frac{\sum_{c=1}^{C}{\mathrm{TP}}_{c}}{N_{\mathrm{total}}},$$

where *C* is the number of classes, ${\mathrm{TP}}_{c}$ denotes the number of correctly classified observations belonging to class *c*, and $N_{\mathrm{total}}$ is the total number of observations in the corresponding evaluation subset.

For each class, recall was calculated as

(20) $${\mathrm{Recall}}_{c}=\frac{{\mathrm{TP}}_{c}}{{\mathrm{TP}}_{c}+{\mathrm{FN}}_{c}},$$

where ${\mathrm{FN}}_{c}$ denotes the number of observations belonging to class *c* that were assigned to another class.

The F1-score for each class was calculated directly from the corresponding true-positive, false-positive, and false-negative counts:

(21) $$F1_{c}=\frac{2{\mathrm{TP}}_{c}}{2{\mathrm{TP}}_{c}+{\mathrm{FP}}_{c}+{\mathrm{FN}}_{c}},$$

where ${\mathrm{FP}}_{c}$ denotes the number of observations from other classes that were incorrectly assigned to class *c*.

The macro-averaged F1-score was calculated as the unweighted mean of the class-wise F1-scores:

(22) $$\mathrm{Macro}\text{-}F1=\frac{1}{C}\sum_{c=1}^{C}F1_{c}.$$

This metric assigns the same importance to all classes, independently of the number of observations contained in each class.

Balanced accuracy was calculated as the unweighted mean of the class-wise recalls:

(23) $$\mathrm{Balanced}\;\mathrm{Accuracy}=\frac{1}{C}\sum_{c=1}^{C}{\mathrm{Recall}}_{c}.$$

Balanced accuracy therefore provides a performance measure that is less affected by differences in class frequency than overall accuracy.

Confusion matrices were used to examine the distribution of predictions among the evaluated classes. In the original sample-level analysis, the matrix included the predefined pedestrian movement classes and the non-step class. In the event-level directional analysis, the matrix was constructed for the corresponding event-level classes. Each row represents the true class, whereas each column represents the predicted class. Diagonal elements correspond to correctly classified observations, while off-diagonal elements indicate misclassifications between classes.

For the original sample-level evaluation, the reported metrics correspond to a single final evaluation on the 20% sample-level hold-out subset and therefore do not represent variability across repeated dataset partitions. For the leakage-aware event-level evaluation, performance is reported as the mean and standard deviation across the five validation folds. When a confusion matrix is presented for an event-level task, the out-of-fold predictions from the five folds are pooled so that each physical footstep contributes exactly once to the matrix. Because the numerical results reported in the tables correspond to the arithmetic mean of the fold-wise metrics, values calculated directly from the pooled out-of-fold confusion matrix may differ slightly from the reported fold means.

### 2.11. Video-Based Labeling and Privacy Considerations

The experimental sequence was recorded on video to facilitate the synchronization and labeling of the pedestrian movements. The video recording was used exclusively as a reference for identifying the movement performed at each time interval and was not used as an input to the classification model.

The proposed sensing method itself relies only on floor-vibration measurements acquired by the triaxial MEMS accelerometer. It does not require facial images, speech recordings, wearable devices, or personal radio identifiers. Consequently, the operational sensing principle is device-free and does not directly capture information that could visually identify the monitored pedestrian.

## 3. Results

This section presents the vibration signals acquired during the experimental campaign and the classification results obtained with the proposed AI-enhanced sensing approach. The results are organized around the primary leakage-aware event-level evaluation, in which complete footsteps constitute the withheld classification units. The original sample-level result is reported separately as a legacy within-sequence reference to document the initial methodology and the effect of temporal dependence in densely sampled observations. All claims regarding directional sensing in this manuscript are based on the event-level analysis.

### 3.1. Measured Floor-Vibration Response

**Figure 7:**
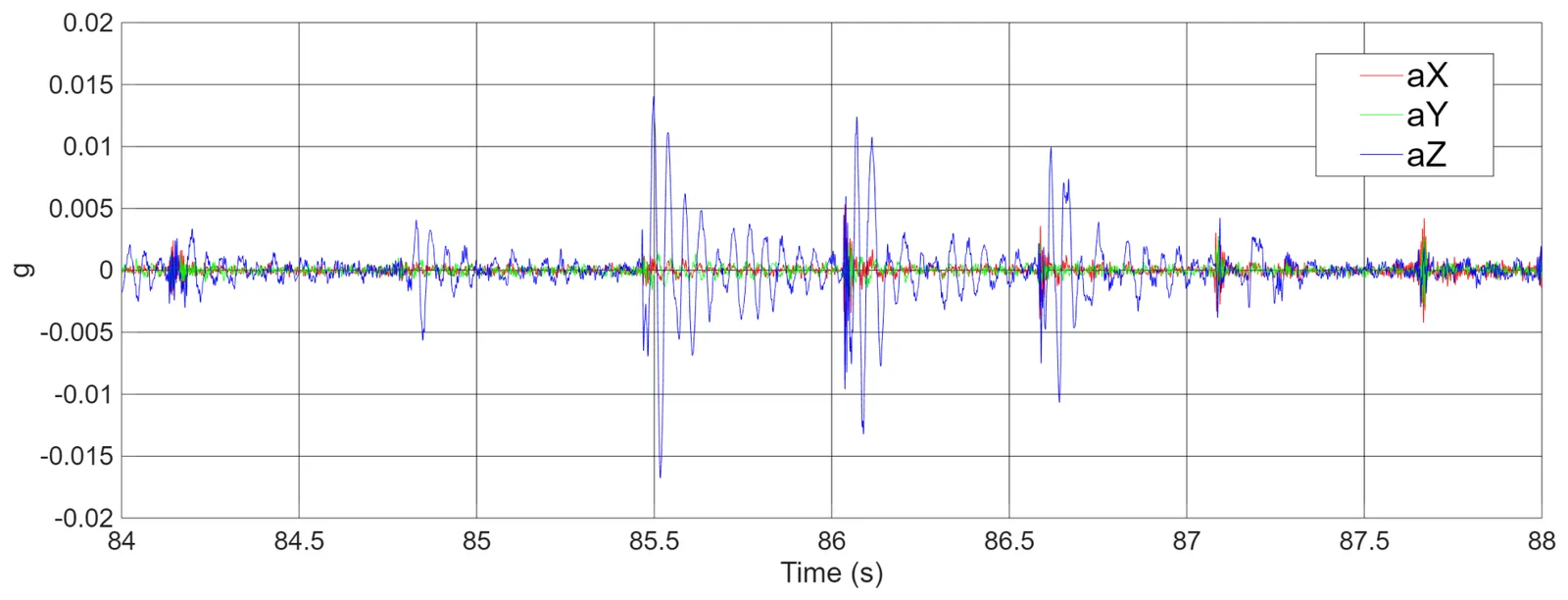
Representative *x*-, *y*-, and *z*-axis acceleration signals recorded between 84 and 88 s of the experimental sequence.

Figure 7 shows a representative segment of the triaxial acceleration signals recorded during the experiment. The selected interval corresponds to the time range between 84 and 88 s and includes several pedestrian footsteps performed in the vicinity of the sensor.

The acquired signals exhibit transient peaks followed by damped oscillations, which are consistent with the structural response generated by foot–floor impacts. The strongest response is generally observed along the *z*-axis, which is approximately normal to the floor surface. Nevertheless, measurable vibration components are also present along the two horizontal axes. The relative amplitude and temporal evolution of the three acceleration components vary among footstep events. These differences indicate that the triaxial structural response contains movement-dependent information. The following subsections investigate to what extent this information can be used for classification under the primary event-level validation, while the original sample-level result is reported separately as a within-sequence reference.

#### Experimental Background-Noise Results

Figure 5 shows the experimental background ASD obtained from the seven inter-step intervals described in Section 2.6. At each frequency, the solid curves represent the mean ASD across the seven intervals, while the shaded regions indicate the interquartile range. The band-limited RMS values reported below were calculated independently for each interval and are summarized as the mean ± standard deviation across the seven interval-wise RMS estimates. The vertical dashed line marks 80 Hz, corresponding to the effective reference bandwidth used in the preliminary noise-design calculation of Section 2.3.2.

Integration of the measured PSD over the 1–80 Hz range resulted in background RMS accelerations of

(24) $$a_{x,\mathrm{RMS}}=127\pm 16\;\mu g,\;a_{y,\mathrm{RMS}}=134\pm 30\;\mu g,\;a_{z,\mathrm{RMS}}=420\pm 194\;\mu g,$$

where the reported values correspond to the mean and standard deviation across the seven background intervals. The X- and Y-axis values remained below the $226\;\mu g_{\mathrm{RMS}}$ reference level derived in Section 2.3.2. In contrast, the Z-axis exhibited a larger and substantially more variable background response.

The corresponding broadband RMS values were 535 ± 119 μg, 506 ± 144 μg, and 894 ± 295 μg for the X-, Y-, and Z-axes, respectively. The higher broadband values experimentally confirm that the total integrated background level depends strongly on the considered bandwidth and that the approximately 80-Hz bandwidth adopted in the design-stage calculation should not be interpreted as the physical acquisition bandwidth used in the experiments.

Inspection of the Z-axis spectrum further showed that its elevated 1–80 Hz RMS value was not associated with a uniformly increased broadband noise floor. Instead, the vertical background response was dominated by recurring low-frequency components, particularly within approximately 10–30 Hz, with prominent spectral maxima around 14–22 Hz. Approximately 83% of the measured Z-axis background power within 1–80 Hz was concentrated in this 10–30 Hz range. This spectral concentration is consistent with structural or environmental vibration transmitted through the floor and the sensor mounting, rather than with accelerometer self-noise alone.

These measurements therefore distinguish the intrinsic design-stage sensor-noise criterion from the experimentally observed background vibration of the installed sensing system. The latter includes the mechanical response of the supporting floor, which is itself part of the measurement chain in vibration-based pedestrian sensing.

### 3.2. Primary Event-Level Performance

The primary classification analysis used complete footsteps as the event-level partitioning units. The event-level evaluation used the 118 manually identified and labeled footsteps available in the dataset. Unlike the original sample-level analysis, the non-step intervals were not included in this evaluation because the released event annotations identify the movement class only for the detected footstep events. As described in Section 2.7, three event-level feature representations were evaluated. The temporally ordered representation $z_{e}$ contains the 15 original lightweight features evaluated over ten successive 25 ms intervals and therefore has 150 dimensions. The compact event descriptor vector $d_{e}$ contains 12 time-domain descriptors derived from RMS acceleration, maximum absolute acceleration, peak-to-peak amplitude, and kurtosis for the three axes. Finally, the combined representation $u_{e}$ concatenates both representations and contains 162 dimensions. The 118 labeled footstep events were distributed among the original movement classes as follows: A (11), B (7), C (10), D (6), E (28), F (12), G (5), H (5), I (8), J (7), and K (19). This class imbalance motivates the use of balanced accuracy and macro-averaged F1-score in addition to overall accuracy.

Table 4 summarizes the event-level performance of the original A–K movement-label task. This analysis is retained to connect the primary event-level formulation with the labels used in the original experimental design; it is not the principal directional task considered in this study.

The event-level results are substantially lower than those obtained in the original sample-level evaluation. In particular, balanced accuracy decreases from 96.37% in the within-sequence sample-level analysis to approximately 17–18% when validation is performed on complete footstep events that have contributed no information to the corresponding training folds. For reference, the balanced chance level for an eleven-class problem is approximately 9.09%. The event-level results therefore indicate that the individual footstep responses retain some discriminative information but that reliable discrimination among the complete A–K movement set cannot be demonstrated from withheld footstep events using the available dataset. This result also indicates that the original geometric labels constitute a more demanding classification problem than the functional directional quantities targeted by the sensing concept. Several linear classes correspond to the same dominant path orientation or travel sense while differing in their position relative to the sensor. The two directional quantities were therefore analyzed separately.

### 3.3. Path-Orientation and Travel-Sense Analysis

The linear movement classes were regrouped according to the two directional quantities defined in the methodology: path orientation and travel sense. Classes A, B, I, and J correspond to movement along the X-orientation, whereas C, D, G, and H correspond to movement along the Y-orientation. Similarly, A, C, G, and J correspond to negative travel sense, whereas B, D, H, and I correspond to positive travel sense. Circular movements E and F were excluded because a complete circular passage has neither a unique global X/Y path orientation nor a unique positive/negative travel sense. The heterogeneous class K was also excluded because it contains indeterminate or transitional movements. Consequently, the functional directional analysis was intentionally restricted to the 59 labeled footsteps belonging to the eight linear trajectory classes. The resulting subset contained 59 labeled linear footsteps. In addition to evaluating path orientation and travel sense independently, a four-direction problem was constructed by grouping the original linear classes as

(25) $$\begin{array}{cc} A+J & \to -X,\\ B+I & \to +X,\\ C+G & \to -Y,\\ D+H & \to +Y. \end{array}$$

Table 5 compares the three event-level feature representations for the path-orientation, travel-sense, and four-direction classification tasks.

**Figure 8:**
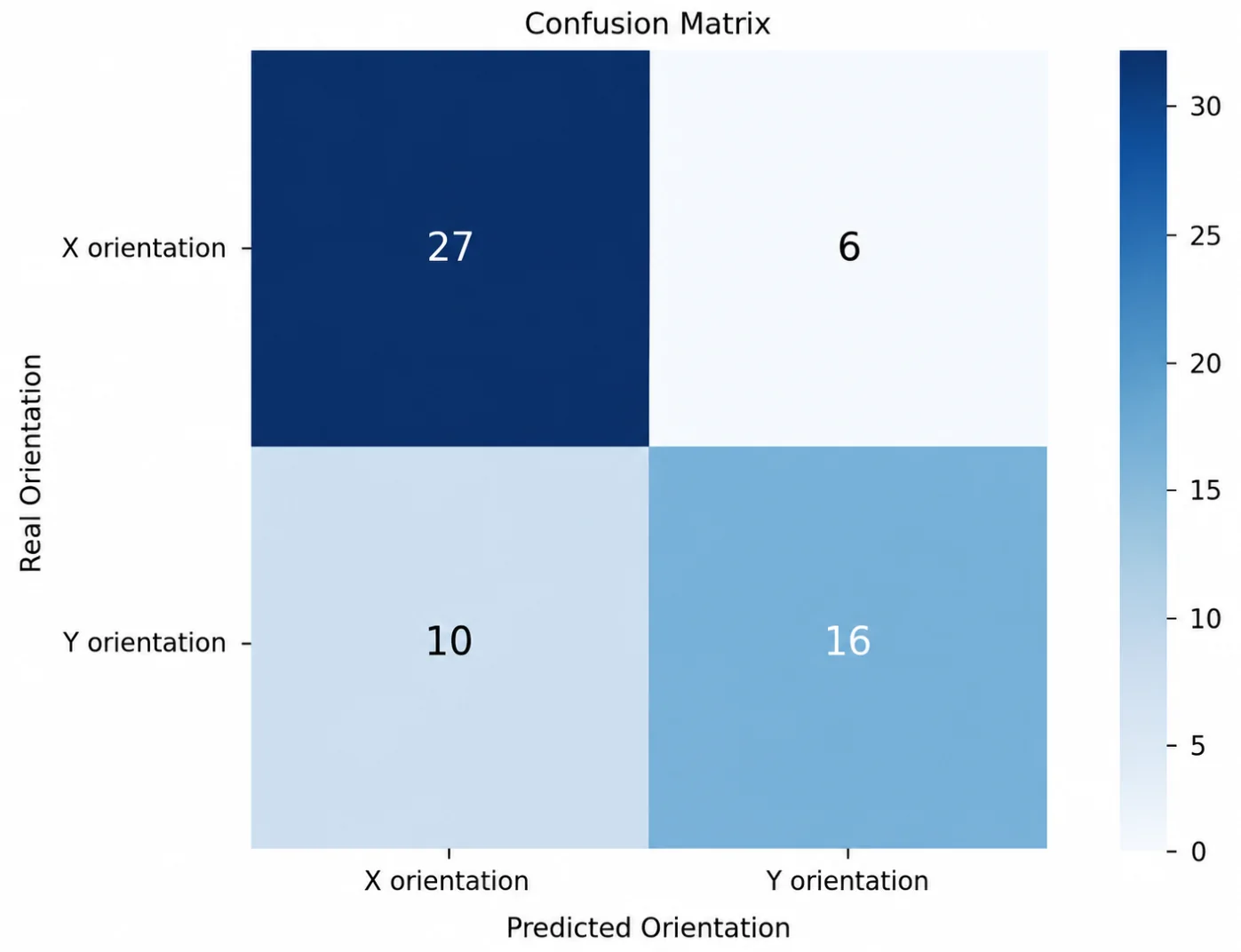
Aggregated out-of-fold confusion matrix for leakage-aware event-level X/Y path-orientation classification using the 12-dimensional temporal descriptor representation $d_{e}$. Predictions from the five validation folds are combined so that each physical footstep event contributes exactly once to the matrix.

A clear difference is observed between the two directional quantities. For path-orientation classification, the compact 12-dimensional event descriptor representation $d_{e}$ achieved the highest performance, with an accuracy of 73.18%, a balanced accuracy of 71.52%, and a macro-F1 score of 70.98%. Figure 8 shows the aggregated out-of-fold confusion matrix obtained for this configuration. Each of the 59 linear footstep events appears exactly once in the matrix, corresponding to the validation fold in which it was withheld from model training.

These results indicate that the local vibration response of an individual footstep contains discriminative information related to whether the pedestrian movement is predominantly aligned with the *X*- or *Y*-orientation. In contrast, none of the evaluated representations reliably distinguished positive from negative travel sense at the level of an isolated footstep. The highest balanced accuracy was 47.00%, which is approximately the 50% chance level for the binary problem. Consistently, the combined four-direction task (+X, −X, +Y, and −Y) also remained difficult. Its highest balanced accuracy was 29.58%, compared with a balanced chance level of 25%. The results therefore indicate that path orientation and travel sense are not equivalently represented in an isolated footstep response. Path orientation is partly encoded in the local triaxial vibration signature, whereas travel sense appears to require information extending beyond a single footstep, such as the evolution of the vibration response across consecutive footsteps along the trajectory.

### 3.4. Axis and Temporal-Descriptor Ablation

An explicit ablation analysis was performed to quantify the contribution of the individual acceleration components and the event-level temporal descriptors. The path-orientation task was selected for this analysis because it was the directional quantity that showed clear above-chance event-level classification performance. First, the 12-dimensional event descriptor representation $d_{e}$ was evaluated using each accelerometer axis individually, all two-axis combinations, and the complete triaxial representation. The same five-fold event-level validation procedure was used in all cases. Table 6 summarizes the results.

The discriminative contribution of the acceleration components was not uniform. The *X*-axis descriptors alone produced performance close to the 50% chance level, whereas the *Y*-axis alone achieved a balanced accuracy of 71.62%. The *Z*-axis also contained useful orientation information, although its performance was lower than that of the *Y*-axis. Combining additional axes did not produce a consistent improvement over the *Y*-axis representation. In particular, the complete XYZ descriptor vector achieved a balanced accuracy of 71.52%, which is essentially equivalent to the result obtained using the *Y*-axis alone. Under the evaluated sensor orientation and structural configuration, orientation information was therefore not distributed equally among the three measured components. A second ablation analysis examined the four event-level descriptors using the *Y*- and *Z*-acceleration components. These two axes were selected because the preceding axis-ablation analysis showed that both retained above-chance path-orientation information when evaluated individually, whereas the *X*-axis alone performed close to chance level. Individual descriptors and a limited set of simple descriptor combinations were compared rather than performing an exhaustive feature-subset search. The combination of RMS and maximum absolute amplitude achieved an accuracy of 73.18%, a balanced accuracy of 72.10%, and a macro-F1 score of 72.18% using only four descriptors,

(26) $$[a_{y,\mathrm{RMS}},a_{z,\mathrm{RMS}},a_{y,\max},a_{z,\max}].$$

For comparison, RMS alone achieved a balanced accuracy of 65.33%, maximum absolute amplitude 64.33%, peak-to-peak amplitude 63.52%, and kurtosis 62.86%. Using all four descriptors from the *Y*- and *Z*-components resulted in a balanced accuracy of 69.86%. The differences among the best-performing configurations are small relative to the fold-to-fold variability and should therefore not be interpreted as evidence that the four-feature subset is statistically superior. Instead, the ablation suggests that a substantial fraction of the path-orientation information observed in the present dataset can be retained using a very small set of low-complexity amplitude descriptors.

### 3.5. Comparison with Lightweight Classifiers

To determine whether the performance obtained with the Random Forest depended strongly on the selected classifier, the leakage-aware X/Y path-orientation experiment was repeated using four additional low-complexity classification methods: logistic regression, Linear SVM, linear discriminant analysis (LDA), and a compact MLP with a single eight-neuron hidden layer. The comparison used the same 12-dimensional event-level temporal descriptor representation $d_{e}$, the same 59 labeled linear footstep events, and exactly the same five cross-validation partitions used for the Random Forest evaluation. Training-only normalization was also maintained independently within every fold.

Table 7 summarizes the resulting performance. The Random Forest achieved the highest mean performance for all three evaluation metrics, with an accuracy of 73.18±14.68%, a balanced accuracy of 71.52±15.80%, and a macro-F1 score of 70.98±17.09%.

The comparison indicates that the path-orientation information contained in the compact temporal descriptors is not equally captured by all classifier families. Among the linear classifiers, logistic regression produced performance close to the binary chance level, while Linear SVM and LDA achieved progressively higher balanced accuracies of 56.62% and 63.76%, respectively. However, all three remained below the Random Forest result.

The compact MLP also achieved above-chance discrimination, with a balanced accuracy of 60.86%. Its 12–8–2 architecture contains only a small number of trainable parameters and is therefore compatible with lightweight embedded inference. Nevertheless, under the present event-level validation, it did not improve upon the Random Forest.

Overall, the Random Forest provided the highest mean accuracy, balanced accuracy, and macro-F1 score among the evaluated classifiers. These results support retaining it as the classifier used in the present sensing pipeline. This conclusion is restricted to the evaluated feature representation, dataset, and validation procedure and should not be interpreted as evidence that Random Forest is universally optimal for vibration-based pedestrian sensing.

### 3.6. Original Sample-Level Reference Performance

**Figure 9:**
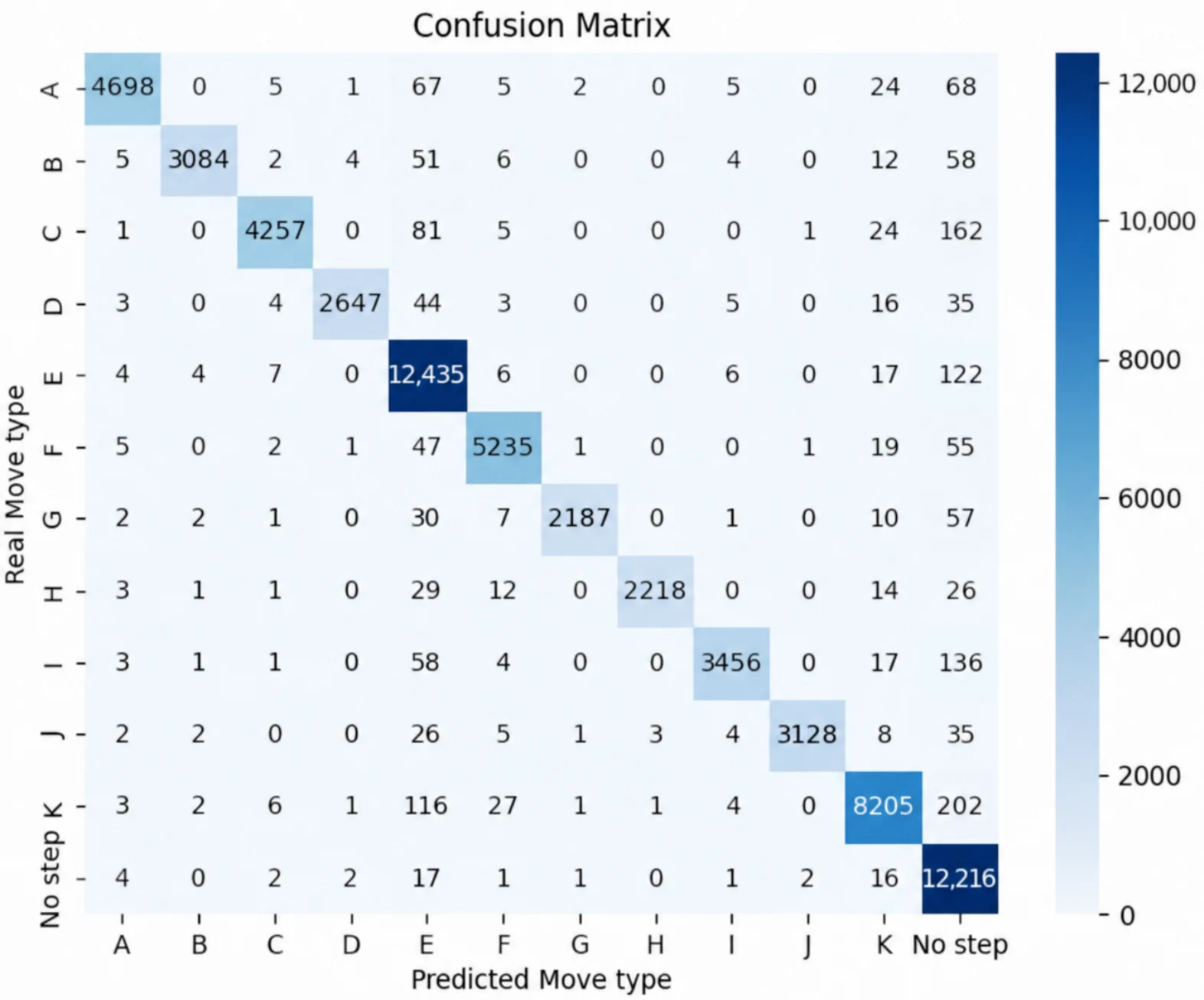
Confusion matrix obtained with the Random Forest classifier under the original within-sequence sample-level evaluation for the predefined pedestrian movements and the non-step class.

For historical comparison with the initial implementation, the original sample-level procedure was reproduced using the 15-dimensional representation composed of the instantaneous *x*-, *y*-, and *z*-axis acceleration values and moving-average features computed with window lengths of 25, 50, 100, and 250 samples. The complete labeled sample-level dataset was divided into an 80% development subset and a 20% sample-level hold-out subset. Five-fold cross-validation was applied within the development subset, after which the classifier was retrained on the complete development data and evaluated on the sample-level hold-out subset. This procedure yielded an overall accuracy of 97.09%, a balanced accuracy of 96.37%, and a macro-averaged F1-score of 97.36%. Figure 9 shows the corresponding confusion matrix for the predefined movement labels and the non-step class.

Most observations are concentrated along the main diagonal, showing a high degree of sample-level separability among the predefined labels within the recorded acquisition sequence. The non-step intervals were also clearly separated from the movement classes under this evaluation procedure. However, this result must be interpreted in the context of the temporal dependence of the observations. Feature vectors were generated at 4 kSPS, whereas the longest moving-average feature contained 250 samples. Consecutive feature vectors could therefore share up to 249 of the 250 raw samples used to calculate the longest temporal feature. Because the original dataset partition was performed at the sample level, strongly overlapping and temporally adjacent observations could be assigned to different subsets. The 97.09% accuracy is therefore retained as a within-sequence sample-level reference performance. It should not be interpreted as an estimate of generalization to complete footsteps as the partitioning units, complete trajectories, participants, measurement sessions, floor structures, or industrial environments.

### 3.7. Implications for AI-Enhanced Sensing

The primary event-level analysis indicates that the strongest directional information supported by the present dataset concerns coarse X-/Y-path orientation. Using a compact set of conventional temporal descriptors, withheld footsteps were classified according to X-/Y-orientation with a balanced accuracy of approximately 72% under the same participant, session, sensor position, and floor structure. In contrast, reliable travel-sense classification could not be demonstrated from isolated footsteps.

The axis and descriptor ablation further indicates that much of this orientation information can be represented using a small number of low-complexity features. Positive and negative displacement along a given orientation appear to require information extending beyond the local vibration response of an isolated event. A future implementation may therefore benefit from a hierarchical approach in which individual footsteps provide local orientation information while travel sense is inferred from the evolution of several consecutive events.

From an embedded-processing perspective, the event-level formulation remains compatible with the original low-complexity objective. Moving averages can be updated incrementally, while RMS, peak-to-peak, maximum amplitude, and similar event descriptors require only simple arithmetic operations. Random Forest inference is based predominantly on comparisons and decision-tree traversal. However, in the present study, the ESP32 was used for signal acquisition and data transmission, whereas feature extraction and classifier inference were performed offline on a computer. The reported results therefore demonstrate the feasibility of extracting path-orientation information from a single floor-mounted triaxial MEMS accelerometer under the evaluated conditions, but they do not yet constitute an on-device Edge AI implementation or demonstrate generalization to unseen participants, acquisition sessions, floor structures, or realistic industrial disturbances.

## 4. Discussion

The primary leakage-aware event-level analysis provides a restricted but more defensible assessment of the directional information contained in footstep-induced floor vibrations. When complete footsteps were used as the data-partitioning units, classification of the original A–K movement-label task was difficult, with a best balanced accuracy of 17.94%. This result indicates that the original detailed geometric labels are not reliably discriminated from withheld footsteps using the present dataset.

A clearer result emerged when the eight linear trajectory classes were regrouped according to the two functional directional quantities defined in this study. Individual footsteps retained measurable information about X-/Y-path orientation, reaching a balanced accuracy of 71.52% with the compact 12-dimensional event-level descriptor representation. In contrast, positive and negative travel sense could not be reliably discriminated from isolated footsteps, with the best balanced accuracy remaining close to the 50% chance level.

The principal finding is therefore deliberately limited: under one participant, one acquisition session, one sensor position, and one floor structure, withheld footstep events contain above-chance information about coarse X-/Y-path orientation. The present experiment does not demonstrate generalization to independent trajectory repetitions, participants, sessions, or structures, and it does not demonstrate reliable travel-sense estimation.

For completeness, the original sample-level procedure produced 97.09% accuracy. This value is retained only as a legacy within-sequence reference and is not used to support the directional-sensing conclusions because densely sampled observations contain strongly overlapping temporal information.

### 4.1. Interpretation of the Directional Information

Footstep-induced vibrations are generated by the interaction between the pedestrian and the supporting floor. Their measured characteristics depend on the applied force, pedestrian trajectory, distance from the sensor, and dynamic response of the structure [15,16,17,18]. The floor therefore acts not only as a propagation medium but also as part of the sensing system.

The event-level results indicate that some properties of pedestrian motion are represented more robustly in the local structural response than others. In particular, the *X*-/*Y*-path-orientation task achieved a balanced accuracy of 71.52% using the 12-dimensional descriptor vector $d_{e}$, whereas the temporally ordered 150-dimensional representation $z_{e}$ achieved 62.14%. Combining both representations into the 162-dimensional vector $u_{e}$ did not improve performance, yielding a balanced accuracy of 65.71%.

This behavior suggests that the global amplitude and impulsiveness characteristics of the complete footstep event provide more stable information about path orientation than the higher-dimensional temporal representation considered here. The result should not be interpreted as evidence that temporal evolution is irrelevant in general; rather, for the available number of withheld footstep events, increasing the feature dimension did not translate into improved event-level discrimination.

The axis-ablation analysis provides additional insight into the measured directional information. The contribution of the three acceleration components was clearly non-uniform. The *X*-axis descriptors alone produced a balanced accuracy of 50.95%, close to chance level, whereas the *Y*-axis alone reached 71.62%. The *Z* component also retained useful information, with a balanced accuracy of 64.19%. Adding additional axes did not provide a consistent improvement over the *Y* component alone, and the complete XYZ representation achieved a similar balanced accuracy of 71.52%.

These results indicate that the information relevant to path orientation is not necessarily distributed equally among the three sensor axes. However, the stronger contribution of the *Y* component should not be interpreted as a general property of footstep vibrations. The relationship between the sensor axes, pedestrian trajectories, floor geometry, structural modes, and sensor position is specific to the present experimental configuration. Different sensor orientations or floor structures may therefore lead to a different distribution of discriminative information among the measured components.

The experimental background-noise characterization provides an additional indication of this axis dependence. In particular, the Z-component exhibited stronger background vibration dominated by low-frequency structural components around 10–30 Hz, whereas the X- and Y-components showed substantially lower 1–80 Hz RMS levels. This observation reinforces the interpretation of the floor structure and sensor coupling as integral parts of the sensing system rather than treating the measured signal as the response of the MEMS device alone.

The temporal-descriptor ablation leads to a similar conclusion regarding feature complexity. The combination of RMS and maximum absolute amplitude from the *Y*- and *Z*-components retained a balanced accuracy of 72.10% using only four descriptors. However, the differences among the best-performing descriptor subsets were small relative to the variability observed across folds. The four-feature configuration should therefore not be interpreted as statistically superior to the complete descriptor set. Instead, the result indicates that a substantial fraction of the path-orientation information can potentially be represented using a small number of low-complexity amplitude features.

A different behavior was observed for travel sense. None of the evaluated event-level representations reliably distinguished positive from negative displacement along a given path orientation. The best balanced accuracy was 47.00%, and the corresponding four-direction problem (+X, −X, +Y, and −Y) reached only 29.58%, compared with a balanced chance level of 25%.

This difference between path orientation and travel sense is physically plausible. An isolated footstep provides a local excitation of the floor and may contain information related to the dominant structural response along a given geometrical orientation. In contrast, distinguishing whether the pedestrian is progressing in the positive or negative sense along that path may depend more strongly on how the response evolves across successive footsteps, for example, through changes associated with relative position and distance from the sensor. The present results therefore suggest that travel-sense estimation should be formulated as a multi-event or sequential problem rather than as the classification of an isolated footstep.

### 4.2. Comparison with Existing Pedestrian-Monitoring Approaches

The proposed sensing concept occupies an intermediate position between conventional occupancy sensors and more complex distributed vibration-monitoring systems. PIR and CO_2_ sensors are simple and privacy-preserving but generally provide presence or indirect occupancy information rather than detailed pedestrian-flow information [3,6]. Camera-based systems can provide counting, tracking, path orientation, and travel direction but involve privacy, lighting, occlusion, processing, and data-management constraints [3,7].

Previous vibration-based studies have demonstrated footstep detection, pedestrian counting, identification, gait assessment, localization, and trajectory reconstruction [7,9,10,11,12,13,17,18,19,20]. Approaches intended to recover detailed spatial information commonly employ several spatially distributed sensing nodes or exploit richer spatial propagation information [7,8,11,12,21]. These studies demonstrate the substantial localization and tracking capability obtainable from structural-vibration sensing but also highlight the dependence of the measured response on sensor arrangement, propagation through the supporting structure, and the availability of sufficient spatial or training information. Multiple sensing positions provide information that is intrinsically unavailable from a single local measurement but at the cost of increased installation complexity, synchronization requirements, communication overhead, calibration effort, and maintenance.

The present approach uses only one floor-mounted triaxial MEMS accelerometer and therefore addresses a more restricted sensing objective. The leakage-aware results support the extraction of path-orientation information from individual footsteps, but they do not demonstrate continuous two-dimensional localization, trajectory reconstruction, or reliable travel-sense estimation.

Consequently, the proposed system should not be interpreted as a direct replacement for distributed localization or tracking systems. Its potential advantage lies instead in situations where a lower-infrastructure sensor can provide useful coarse directional information, such as distinguishing passages predominantly aligned with two different axes or routes. Functions requiring signed direction, such as distinguishing entry from exit, would require an additional travel-sense estimation stage that has not yet been validated in the present study.

### 4.3. AI Enhancement and Embedded-Sensor Perspective

A conventional accelerometer provides low-level measurements of acceleration along three axes. In the proposed system, temporal feature extraction and machine-learning classification are used to transform these measurements into higher-level information related to pedestrian movement. In this sense, the AI-enhanced functionality lies in the interpretation of the measured structural response rather than in the use of a computationally complex learning architecture.

The revised results reinforce the interest of maintaining a lightweight feature representation. The best event-level path-orientation result was obtained with only 12 conventional temporal descriptors rather than with the 150- or 162-dimensional representations. Moreover, the descriptor ablation indicated that a four-feature subset based on RMS and maximum absolute amplitude retained comparable mean performance under the present validation. The present results show that useful path-orientation information can be extracted using low-dimensional time-domain descriptors. However, the current study does not establish the superiority of these descriptors over frequency-domain or time–frequency representations. Their main advantage in the present context is their low computational complexity, which makes them attractive candidates for future embedded implementation.

The classifier comparison further supports the use of Random Forest in the present classification pipeline. Using identical event-level partitions and the same 12-dimensional descriptor representation, Random Forest achieved higher mean accuracy, balanced accuracy, and macro-F1 than logistic regression, Linear SVM, LDA, and the compact MLP. The result is particularly relevant from an embedded-sensing perspective because the improvement did not require a computationally intensive deep-learning architecture.

At the same time, the comparison shows that useful path-orientation information is not exclusive to the Random Forest model. Linear SVM, LDA, and the compact MLP all retained above-chance discrimination, although with lower mean performance. The comparatively low performance of the linear classifiers, particularly logistic regression and Linear SVM, suggests that the relationship between the selected temporal descriptors and path orientation may not be adequately represented by a simple linear decision boundary under the present experimental conditions. Given the limited number of footstep events, however, differences between classifiers should be interpreted with caution and require confirmation on larger independent datasets.

RMS, maximum amplitude, peak-to-peak amplitude, and moving averages can all be calculated using comparatively simple arithmetic operations, while Random Forest inference consists mainly of threshold comparisons and decision-tree traversal. These characteristics are compatible with the resource constraints of embedded sensing platforms such as the ESP32 used in the acquisition system [25]. Although the computational and memory requirements of the evaluated classifiers were not measured experimentally on the ESP32, all five models were selected as low-complexity candidates for future embedded implementation, and the Random Forest provided the highest classification performance under the present offline evaluation.

Nevertheless, compatibility with embedded execution should be distinguished from demonstrated edge AI operation. In the present implementation, the ESP32 was used for sensor configuration, acquisition, and transmission, whereas feature extraction and Random Forest inference were performed offline on a computer. Memory footprint, processing time, inference latency, power consumption, and sustained real-time operation were not measured. The current study therefore demonstrates the feasibility of the sensing and classification concept but does not yet demonstrate a complete on-device AI implementation.

A future embedded architecture could perform footstep detection and event-level feature extraction locally and transmit only the estimated path orientation, timestamp, confidence value, or other event-level information rather than the complete 4 kSPS triaxial waveform. Such an approach could substantially reduce communication and storage requirements. Travel-sense estimation, if subsequently demonstrated, would probably require maintaining a short sequence of consecutive footstep events rather than processing each event independently.

### 4.4. Potential Industrial Applications

The proposed sensing concept is relevant to human-centric industrial and smart-building environments in which pedestrian information is desirable without the use of cameras or wearable devices. A single compact floor-coupled node could potentially support applications such as monitoring whether pedestrian passages occur predominantly along longitudinal or transverse routes, detecting activity in specific circulation paths, or providing coarse movement information for occupancy-aware building functions.

The single-node architecture may be particularly attractive for retrofit installations, where additional wiring, distributed synchronization, and modification of the existing infrastructure should be minimized. Moreover, the operational sensing principle uses floor vibrations and does not require facial images, speech recordings, wearable devices, or personal radio identifiers.

However, the revised results also define an important boundary for the current applications. Entry/exit discrimination, movement toward or away from a restricted area, and other tasks requiring reliable positive/negative travel sense cannot presently be claimed from individual footsteps. Implementing these functions would require additional temporal information from consecutive footsteps, additional sensing information, or both.

The system has also not been evaluated for simultaneous pedestrians, continuous person counting, crowded environments, or safety-critical decision making. Applications requiring certified detection reliability, individual localization, robust multiperson tracking, or reliable entry/exit decisions would therefore require substantially broader validation and potentially additional sensing redundancy.

### 4.5. Limitations of the Present Study

The principal limitation of the present study is the restricted experimental dataset. All measurements were obtained from a single participant during one continuous acquisition session, using one sensor position and one floor structure. Each predefined trajectory was executed once. Consequently, neither the original sample-level result nor the leakage-aware event-level results can be interpreted as evidence of generalization across participants, sessions, sensor positions, or supporting structures.

The leakage-aware event-level validation addresses one important limitation of the original evaluation by ensuring that complete physical footsteps, rather than strongly overlapping sample-level feature vectors, constitute the classification units. Furthermore, the raw-sample supports associated with different event representations do not overlap. This substantially reduces the direct temporal leakage present in the original sample-level partitioning.

However, event-level independence is not equivalent to trajectory-level or experimental-condition independence. Footsteps from the same continuous trajectory execution can appear in different cross-validation folds. Therefore, the reported event-level performance measures generalization to previously unseen footsteps acquired under the same participant, session, trajectory protocol, sensor position, and floor structure. It does not quantify performance on a completely unseen trajectory execution.

The limited number of labeled footstep events also introduces substantial statistical uncertainty. The complete event-level dataset contains 118 labeled footsteps, and the path-orientation analysis uses only 59 linear footsteps. The relatively large standard deviations observed across the five folds are therefore an important part of the reported result and prevent small differences between feature configurations from being interpreted as evidence of superiority.

The feature analysis was also limited in scope. The revised event-level analysis focused on low-complexity time-domain descriptors, and a systematic comparison with frequency-domain or time–frequency features was not performed. Consequently, the present results should not be interpreted as evidence that the selected temporal descriptors are optimal for this sensing problem. A broader comparison of feature families should be conducted on a larger dataset using predefined validation groups to avoid introducing additional model-selection bias.

A further limitation concerns the event definition. Only the most significant peak of each footstep was manually annotated, whereas exact footstep onset and end times were not available. Fixed 250 ms windows centered on the annotated peaks were therefore used to construct the event-level representations. Although the windows were selected to remain non-overlapping and to capture the local response, alternative event segmentation strategies may affect the extracted descriptors and should be evaluated with more extensively annotated data.

The leakage-aware event-level analysis also focuses exclusively on the 118 labeled footstep events. Non-step intervals are included in the original sample-level analysis but are not part of the revised event-level directional evaluation. Consequently, the event-level results characterize classification after a footstep event has been identified; they do not represent the performance of a complete end-to-end system that must first detect footsteps in continuous data and then infer their directional information.

Floor composition, structural geometry, boundary conditions, sensor coupling, and sensor orientation can substantially modify the measured vibration response [7,15,16,18]. Environmental disturbances such as machinery, doors, mobile equipment, falling objects, chairs, and other pedestrians may also generate transient structural vibrations. These conditions were intentionally minimized in the current controlled experiment and therefore remain outside its validated scope.

The experiment involved only one pedestrian at a time. Overlapping footsteps from several individuals are known to complicate vibration-based monitoring [7,19], and no conclusions regarding multiperson operation can be drawn from the present dataset.

The limited number of events also prevents a rigorous characterization of the classifier bias–variance trade-off. The comparison among Random Forest, logistic regression, Linear SVM, LDA, and the compact MLP used fixed model configurations and identical validation folds, without classifier-specific hyperparameter optimization. It should therefore be interpreted as a baseline classifier comparison rather than as a comprehensive model-selection or bias–variance study.

Finally, the Random Forest configuration was intentionally kept unchanged in the leakage-aware analyses so that the effect of changing the validation unit and feature representation could be examined without simultaneously retuning the classifier. Although Random Forest achieved the highest mean performance among the five lightweight classifiers evaluated here, the limited dataset and the restricted set of classifier families considered do not establish Random Forest as universally optimal for the event-level problem.

### 4.6. Future Development

Future work should first extend the experimental dataset to include multiple participants, repeated acquisitions of the same trajectories, different walking speeds and footwear, several sensor positions, and heterogeneous floor structures. Repeated sessions are particularly important because they would permit trajectory-wise, session-wise, and participant-wise evaluation using genuinely independent experimental groups.

Realistic background disturbances should also be incorporated, including machinery, doors, mobile equipment, object impacts, and simultaneous pedestrians. This would permit the complete sensing chain to be evaluated from continuous vibration recordings, including footstep detection, directional classification, rejection of unknown events, and false-alarm performance.

The present distinction between path orientation and travel sense suggests a natural hierarchical processing strategy. Individual footstep events could first be used to estimate local path orientation, while a second stage could integrate several consecutive events to infer travel sense from their temporal progression. Such a formulation should be evaluated using complete trajectory repetitions as independent units rather than distributing individual footsteps from the same passage across folds.

The event-level feature analysis should also be repeated on larger datasets to determine whether the compact amplitude descriptors remain competitive across participants and structures. The axis dependence observed in the present experiment should likewise be reassessed after changing the sensor orientation and supporting floor, since the relative contribution of the three acceleration components is expected to depend on the structural configuration.

The lightweight-classifier comparison should be repeated on larger and independently grouped datasets to determine whether the relative advantage observed for Random Forest persists across participants, sessions, and floor structures.

Adaptive processing may ultimately be required to transfer the system between structures. Possible approaches include structure-specific calibration, adaptive filtering, domain adaptation, physics-informed features, or hybrid methods that combine structural information with data-driven classification.

Finally, feature extraction and Random Forest inference should be implemented directly on the ESP32 or on a comparable embedded platform. The resulting system should be characterized in terms of memory use, processing time, inference latency, power consumption, communication load, and sustained real-time operation. This step would allow the proposed architecture to be evaluated as a complete embedded AI-enhanced sensing node rather than as an offline proof of concept.

## 5. Conclusions

This study investigated whether footstep-induced structural vibrations measured with a single floor-mounted triaxial MEMS accelerometer contain information related to pedestrian movement orientation. The primary evaluation used leakage-aware event-level cross-validation, with complete footsteps constituting the data-partitioning units.

For the original A–K movement-label task, classification of withheld footsteps remained difficult. However, when the linear trajectories were regrouped according to the functional directional quantities defined in the study, individual footsteps retained measurable information about coarse X-/Y-path orientation. Using the compact 12-dimensional event-level descriptor representation, Random Forest classification achieved an accuracy of 73.18%, a balanced accuracy of 71.52%, and a macro-F1 score of 70.98%. Axis and descriptor ablation further indicated that a substantial fraction of this information can be represented using a small number of low-complexity amplitude descriptors.

In contrast, reliable positive/negative travel-sense discrimination could not be demonstrated from isolated footsteps. The present results therefore support only the more restricted conclusion that coarse X-/Y-path-orientation information is present in withheld footsteps under the evaluated experimental conditions. They do not demonstrate signed travel direction, continuous trajectory reconstruction, or generalization to independent participants, sessions, trajectory repetitions, sensor positions, or floor structures.

For historical comparison, the original sample-level procedure yielded 97.09% accuracy. This value is retained only as a within-sequence reference and is not used as evidence of generalization because densely sampled feature vectors contain strongly overlapping temporal information. These findings provide proof-of-concept evidence that a single floor-mounted MEMS accelerometer can contain usable information about coarse pedestrian path orientation under controlled conditions.

The present results remain limited to one participant, one acquisition session, one sensor position, and one floor structure. Event-level cross-validation prevents direct sharing of footstep representations and raw samples between training and validation folds, but it does not establish generalization across complete trajectory repetitions, participants, sessions, or structures. The event-level analysis also assumes that a footstep event has already been identified and therefore does not constitute an end-to-end evaluation of continuous footstep detection and directional classification.

Future work should consequently extend the dataset to multiple participants, repeated trajectories and acquisition sessions, different floor structures and sensor positions, and realistic environmental disturbances, including simultaneous pedestrians. Travel-sense estimation should be reformulated using sequences of consecutive footsteps and evaluated with complete trajectory repetitions as independent units. Finally, feature extraction and Random Forest inference should be implemented directly on the ESP32 or a comparable embedded platform to quantify memory use, processing latency, power consumption, and real-time operating capability.

## Figures and Tables

**Table 1:** Comparison of sensing technologies for pedestrian-flow monitoring in industrial and smart-building environments.

Technology	Presence	Counting	Path Orientation	Travel Sense	Infrastructure	Main Limitations
PIR sensors [3,6]	Yes	Limited	No	No	Low; wall- or ceiling-mounted sensors	Require motion and provide limited spatial information; stationary occupants may not be detected reliably.
CO_2_ sensors [3,6]	Indirect	Estimated	No	No	Low; environmental sensor	Indirect and slow response; affected by ventilation, room volume, and environmental conditions.
Optical cameras [3,7]	Yes	Yes	Yes	Yes	Medium–high; cameras, communication, and processing	Privacy concerns; sensitivity to lighting and occlusion; substantial processing and data-management requirements.
Pressure mats [3]	Yes	Yes	Possible	Possible	High and localized; floor-integrated installation	Intrusive installation, limited spatial coverage, and reduced suitability for large-scale retrofitting.
Vibration sensing [7,8,9]	Yes	Possible	Possible	Possible	Low–medium; floor-coupled vibration sensors	Affected by floor structure, sensor position, environmental vibrations, and overlapping footsteps.
Proposed approach	Yes	Not evaluated	Yes	Not demonstrated	One floor-mounted triaxial MEMS accelerometer	Evaluated in one controlled environment; generalization across users, structures, and industrial disturbances remains to be assessed.

**Table 2:** Comparison of representative vibration-based pedestrian, occupancy, gait-monitoring, and localization studies.

Reference	Main Task	Sensor Type	Sensors	Processing Approach	Path Orientation	Travel Sense	Main Limitation or Observation
Succi et al. [10]	Footstep detection and approximate tracking	Geophones	–	Amplitude- and temporal-pattern analysis	Possible	Possible	Tracking and bearing estimation were investigated using seismic sensing; the study was not formulated as discrete path-orientation or travel-sense classification.
Pan et al. [19]	Occupant-traffic estimation	Geophones	2	Event extraction, signal-mixture features, and KNN	No	No	Focused on estimating the number of simultaneous pedestrians rather than their movement orientation or travel sense; overlapping footsteps are a central challenge.
Pan et al. [17]	Pedestrian identification	Vibration sensors	Multiple	Physical vibration features and transductive SVM	No	No	Focused on pedestrian identity rather than movement direction; performance is affected by gait variability, step location, and structural heterogeneity.
Poston et al. [11]	Indoor footstep localization	Structural accelerometers	Multiple	Arrival-time estimation and TDoA-based localization	Possible	Possible	Provides footstep positions with sub-meter localization, from which trajectory direction may be derived across consecutive locations; direction is not itself the primary classification task.
Mirshekari et al. [12]	Step-level occupant localization	Floor-vibration sensors	Multiple	Signal decomposition, TDoA, and adaptive multilateration	Possible	Possible	Achieves step-level localization in heterogeneous structures; directional motion would require combining consecutive localized footsteps.
Li et al. [8]	Localization and trajectory tracking	Multicomp. seismometers	Multiple	Angle-constrained TDoA localization and trajectory tracking	Yes	Yes	Provides detailed spatial localization and trajectory reconstruction but requires a distributed multicomponent seismic sensing network.
Drira et al. [7]	Footstep detection, occupant counting, and recognition	Floor-vibration sensor network	Multiple	Event extraction, SVM, and CNN	No	No	Addresses footstep detection, occupant number, and identity; simultaneous walkers and vibration events from other sources complicate the analysis.
Yu et al. [9]	Pedestrian counting	Piezoelectric sensors	Multiple	Spatial-differential vibration features and learning-based counting	No	No	Designed for pedestrian counting rather than path-orientation or travel-sense estimation.
Dong and Noh [18]	Gait-parameter estimation	Geophones	8	Floor-adaptive gait-feature extraction	No	No	Estimates temporal and spatial gait parameters across different floor types but does not address directional pedestrian-flow classification.
Dong et al. [20]	Functional gait assessment	Floor-vibration sensors	Multiple	Hierarchical feature aggregation and neural-network classification	No	No	Designed to infer functional gait stages from sequences of footsteps rather than pedestrian movement direction.
Chakraborty et al. [13]	Structural-vibration gait dataset	Geophone	1	Dataset acquisition for gait-recognition and variability studies	No	No	Includes 100 participants and variation in floor type, sensor distance, and walking speed; focused on gait recognition rather than directional monitoring.
Appelle et al. [21]	Pedestrian localization	Geophones	Multiple	1D-CNN-based TDoA estimation and localization	Possible	Possible	Localizes individuals and small pedestrian groups; movement direction can be derived from successive positions but is not the primary classification output.
Proposed approach	Coarse X/Y path-orientation classification	Triaxial MEMS accelerometer	1	Event-level temporal descriptors and Random Forest classification	Yes	Not demonstrated	Above-chance X/Y path-orientation discrimination was obtained for withheld footstep events under the same participant, session, sensor position, and floor structure; travel sense was not reliably discriminated from isolated footsteps.

**Table 3:** Comparison of vibration transducers for embedded pedestrian-flow sensing.

Sensor Type	Measured Quantity	Typical Useful Range	Signal Conditioning and Interface	Embedded Integration	Main Observation
IEPE accelerometer	Acceleration	Wide bandwidth; typically from a few hertz to several kilohertz	Requires constant-current excitation and analog acquisition	Medium	High sensitivity and low noise, but increased conditioning, power, and system cost.
Geophone	Velocity	Mainly optimized for low-frequency vibration	Analog output requiring amplification and acquisition	Medium	High low-frequency sensitivity, but usually single-axis and less convenient for compact digital integration.
Piezoelectric sensor	Charge or voltage	Well suited to transient and medium-to-high-frequency vibration	Usually requires charge or voltage conditioning	Medium–high	Compact and sensitive to impacts, but directional information generally requires multiple sensing elements.
MEMS accelerometer	Acceleration	DC to several hundred hertz or kilohertz, depending on the device	Digital SPI or I^2^C interface available	High	Compact, low-power, triaxial, and compatible with embedded and IIoT sensing nodes.
ADXL355 used in this study [24]	Triaxial acceleration	Selected for the 1–100 Hz target band; output data rate up to 4 kSPS	Integrated digital conversion and SPI interface	High	Low-noise 20-bit output and direct connection to the ESP32 without external analog conditioning.

**Table 4:** Leakage-aware event-level classification performance for the original A–K movement-label task. Values are reported as mean ± standard deviation across the five folds.

Representation	Dim.	Accuracy (%)	Balanced Acc. (%)	Macro-F1 (%)
$z_{e}$	150	29.75±8.26	17.94±5.58	14.50±4.97
$d_{e}$	12	22.83±11.96	16.88±10.57	14.13±9.72
$u_{e}$	162	30.58±4.20	16.85±3.77	12.90±3.70

**Table 5:** Leakage-aware event-level performance for the functional directional classification tasks. Values are reported as mean ± standard deviation across the five folds.

Task	Representation	Accuracy (%)	Balanced Acc. (%)	Macro-F1 (%)
Path orientation (X/Y)	$z_{e}$	64.39±14.88	62.14±13.76	60.73±15.86
	$d_{e}$	73.18±14.68	71.52±15.80	70.98±17.09
	$u_{e}$	68.03±10.35	65.71±12.32	63.12±17.11
Travel sense (+/−)	$z_{e}$	49.09±15.72	47.00±16.68	43.81±18.09
	$d_{e}$	31.97±14.54	31.43±15.93	30.47±15.72
	$u_{e}$	42.42±6.13	39.81±5.33	37.09±5.66
Four directions	$z_{e}$	28.79±7.27	27.92±7.74	27.09±8.65
	$d_{e}$	28.79±4.25	29.58±5.97	29.12±5.63
	$u_{e}$	20.15±9.27	17.92±8.79	17.67±11.87

**Table 6:** Axis-ablation results for event-level X/Y path-orientation classification using the temporal descriptor representation. Values are means across the five event-level validation folds.

Axes	Accuracy (%)	Balanced Acc. (%)	Macro-F1 (%)
X	50.91	50.95	49.52
Y	73.18	71.62	71.77
Z	66.21	64.19	61.97
XY	71.52	70.19	69.29
XZ	73.03	71.29	68.37
YZ	71.36	69.86	69.88
XYZ	73.18	71.52	70.98

**Table 7:** Comparison of lightweight classifiers for leakage-aware event-level X/Y path-orientation classification using the 12-dimensional temporal descriptor representation $d_{e}$. All classifiers were evaluated using the same 59 labeled linear footstep events, the same five stratified cross-validation folds, and the same training-only normalization procedure. Values are reported as mean ± standard deviation across the five validation folds.

Classifier	Accuracy (%)	Balanced Acc. (%)	Macro-F1 (%)
Random Forest	73.18±14.68	71.52±15.80	70.98±17.09
LDA	64.55±15.70	63.76±16.71	62.43±16.85
Compact MLP (12–8–2)	62.73±4.20	60.86±5.80	59.00±6.61
Linear SVM	59.24±4.46	56.62±4.11	55.24±4.74
Logistic regression	54.24±4.17	50.29±6.11	44.77±6.82

## Data Availability

The data presented in this study are openly available in Zenodo at https://doi.org/10.5281/zenodo.21626419 [26]. The repository contains the raw 4 kSPS triaxial acceleration recordings ($a_{x}$, $a_{y}$, $a_{z}$) together with the corresponding movement annotations, and the video recording used as the reference for manual labeling.

## Abbreviations

The following abbreviations are used in this manuscript:

AI	Artificial Intelligence
ASD	Acceleration Spectral Density
CNN	Convolutional Neural Network
CSV	Comma-Separated Values
DC	Direct Current
I^2^C	Inter-Integrated Circuit
IEPE	Integrated Electronics Piezoelectric
IIoT	Industrial Internet of Things
KNN	K-Nearest Neighbors
kSPS	Kilosamples Per Second
LDA	Linear Discriminant Analysis
LSB	Least Significant Bit
MEMS	Micro-Electro-Mechanical System
MLP	Multilayer Perceptron
PCB	Printed Circuit Board
PIR	Passive Infrared
PSD	Power Spectral Density
RMS	Root Mean Square
SPI	Serial Peripheral Interface
SRAM	Static Random-Access Memory
SVM	Support Vector Machine
UART	Universal Asynchronous Receiver-Transmitter
USB	Universal Serial Bus
